# Plasma metabolites associated with colorectal cancer: A discovery‐replication strategy

**DOI:** 10.1002/ijc.32146

**Published:** 2019-02-14

**Authors:** Anne J.M.R. Geijsen, Stefanie Brezina, Pekka Keski‐Rahkonen, Andreas Baierl, Thomas Bachleitner‐Hofmann, Michael M. Bergmann, Juergen Boehm, Hermann Brenner, Jenny Chang‐Claude, Fränzel J.B. van Duijnhoven, Biljana Gigic, Tanja Gumpenberger, Philipp Hofer, Michael Hoffmeister, Andreana N. Holowatyj, Judith Karner‐Hanusch, Dieuwertje E. Kok, Gernot Leeb, Arve Ulvik, Nivonirina Robinot, Jennifer Ose, Anton Stift, Petra Schrotz‐King, Alexis B. Ulrich, Per Magne Ueland, Ellen Kampman, Augustin Scalbert, Nina Habermann, Andrea Gsur, Cornelia M. Ulrich

**Affiliations:** ^1^ Division of Human Nutrition and Health Wageningen University & Research Wageningen The Netherlands; ^2^ Institute of Cancer Research, Department of Medicine I Medical University of Vienna Austria; ^3^ Biomarkers Group International Agency for Research on Cancer Lyon France; ^4^ Department of Statistics and Operations Research University of Vienna Austria; ^5^ Department of Surgery Medical University Vienna Austria; ^6^ Huntsman Cancer Institute Salt Lake City UT; ^7^ Department of Population Health Sciences University of Utah Salt Lake City UT; ^8^ Division of Preventive Oncology National Center for Tumor Diseases and German Cancer Research Center Heidelberg Germany; ^9^ Division of Clinical Epidemiology and Aging Research German Cancer Research Center (DKFZ) Heidelberg Germany; ^10^ German Cancer Consortium (DKTK) German Cancer Research Center (DKFZ) Heidelberg Germany; ^11^ Division of Cancer Epidemiology German Cancer Research Center Heidelberg Germany; ^12^ Department of General, Visceral and Transplantation Surgery University of Heidelberg Germany; ^13^ Hospital Oberpullendorf Burgenland Austria; ^14^ BEVITAL Bergen Norway; ^15^ Genome Biology European Molecular Biology Laboratory (EMBL) Heidelberg Germany

**Keywords:** colorectal cancer, metabolomics, discovery‐replication approach, UHPLC‐QTOF‐MS

## Abstract

Colorectal cancer is known to arise from multiple tumorigenic pathways; however, the underlying mechanisms remain not completely understood. Metabolomics is becoming an increasingly popular tool in assessing biological processes. Previous metabolomics research focusing on colorectal cancer is limited by sample size and did not replicate findings in independent study populations to verify robustness of reported findings. Here, we performed a ultrahigh performance liquid chromatography‐quadrupole time‐of‐flight mass spectrometry (UHPLC‐QTOF‐MS) screening on EDTA plasma from 268 colorectal cancer patients and 353 controls using independent discovery and replication sets from two European cohorts (ColoCare Study: n = 180 patients/n = 153 controls; the Colorectal Cancer Study of Austria (CORSA) n = 88 patients/n = 200 controls), aiming to identify circulating plasma metabolites associated with colorectal cancer and to improve knowledge regarding colorectal cancer etiology. Multiple logistic regression models were used to test the association between disease state and metabolic features. Statistically significant associated features in the discovery set were taken forward and tested in the replication set to assure robustness of our findings. All models were adjusted for sex, age, BMI and smoking status and corrected for multiple testing using False Discovery Rate. Demographic and clinical data were abstracted from questionnaires and medical records.

## Abstract 2

**What's new?**

Colorectal cancer exhibits certain characteristic changes in metabolic pathways. To expand upon previous findings, these authors performed a discovery‐replication study using two large independent study populations from different countries, Germany and Austria. They tested metabolic profiles of cancer patients and controls, identifying 691 statistically significant features in the discovery cohort. Testing the second cohort narrowed it to 97. These corresponded to 28 metabolites, of which 15 could be identified. It will be useful to go forward with prospective analysis on these 15 metabolites, to determine whether they have predictive or prognostic value.

**Abbreviations**
BCAA: branched chain amino acid
BMI: body mass index
CI: confidence interval
CORSA: Colorectal Cancer Study of Austria
ESI: electrospray ionization
FDR: false discovery rate
FIT: fecal Immunochemical Testing
IARC: International Agency for Research on Cancer
IQR: interquartile range
LysoPEs: lysophosphatidylethanolamines
LysoPCs: lysophosphatidylcholines
MNA: 1‐methylnicotinamide
MS: mass spectrometry
MSI: the Metabolomics Standards Initiative
OR.std: log standardized odds ratios
(Q)TOF: (Quadrupole) time‐of‐flight
SD: standard deviation
TCA: tricarboxylic acid
UHPLC: ultrahigh performance liquid chromatography
WHO: World Health Organization

## Background

Colorectal cancer is a major public health concern worldwide, with 1.4 million new cases and an estimated 700,000 deaths annually.^1^ Colorectal cancer is characterized by a distinct metabolic phenotype and changes in key metabolic pathways such as glycolysis or the tricarboxylic acid (TCA) cycle.^2^, ^3^ Yet, underlying mechanisms involved in colorectal carcinogenesis are still unclear^4^.

Metabolomics is a powerful approach to unravel metabolic changes associated with disease and is gaining momentum in the field of cancer epidemiology.^5^, ^6^, ^7^ Compared to other “‐omics” techniques, metabolomics is more closely related to a measured clinical phenotype and is increasingly applied as the method of choice to screen for potential metabolites associated with disease status.^8^, ^9^ Moreover, metabolomics can help to understand the underlying etiology of cancer development.^10^

Differences in metabolic profiles have been reported between colorectal cancer patients and colorectal cancer‐free individuals using nuclear magnetic resonance techniques,^11^ gas chromatography,^12^, ^13^, ^14^, ^15^ and liquid chromatography‐mass spectrometry methods.^16^

Various amino acids, such as aspartic acid, have been shown to be more abundant in cases in different, relatively small, studies, including a study by Nishiumi and colleagues comparing serum metabolite levels of 60 colorectal cancer patients and 60 healthy volunteers using gas‐chromatography time‐of‐flight (TOF) mass spectrometry.^13^ Similarly, a study by Denkert *et al*. examined metabolic profiles in colon tissue and normal mucosa samples of 27 colorectal cancer patients and 18 colorectal cancer‐free individuals.^15^ In addition to amino acids, serum taurine was shown to be more abundant among colorectal cancer patients compared to colorectal cancer‐free individuals. Another study among 101 newly diagnosed colorectal cancer patients reported a clear difference between serum glutamine, fatty acids, and the urea and TCA cycle metabolites compared to 102 colorectal cancer‐free controls.^17^

The majority of these previous studies have been limited by sample size and did not perform replication of their findings in independent study populations. As metabolomics studies often identified a wide range of metabolites due to the variety of analytical platforms, clinical protocols, and sample handling procedures used, leveraging an independent population for replication using the same platform and similar protocols is essential to ensure robustness of findings. To date, only few studies have used a discovery‐replication design to reproduce results in independent study populations.^16^, ^18^, ^19^ Two of these studies investigated metabolic differences between colorectal cancer patients and apparently healthy individuals;^16^, ^18^ a third study evaluated metabolomic differences between matched tumor and healthy colon tissue samples from colorectal cancer patients.^19^ In addition, a very recent study investigating metabolic profiles in adenomas, colorectal cancer cases and controls conducted analysis in two datasets utilizing different metabolomic approaches, but with both sample sets deriving from the same hospital and cohort.^20^

To complement current research, we utilized a powerful combination of untargeted metabolomics analysis, able to reveal (novel) metabolites, a rigorous discovery‐replication design, leveraging samples deriving from two independent study populations, as well as relatively large sample sizes to obtain sufficient statistical power. The overall purpose of our study was to discover, and replicate plasma metabolites associated with colorectal cancer to improve knowledge regarding potential disease etiology.

## Methods

### Study populations

We utilized data from two cohort studies embedded in the MetaboCCC Consortium, a consortium of four independent European cohorts to investigate metabolic profiles across the continuum of colorectal carcinogenesis: (1) the Heidelberg site of the international ColoCare Study (ClinicalTrials.gov Identifier: NCT02328677) and (2) the Colorectal Cancer Study of Austria (CORSA). The CORSA and ColoCare studies were selected given the availability of samples from colorectal cancer patients as well as controls. EDTA plasma samples from 621 participants were analyzed, consisting of 268 patients with newly diagnosed colorectal cancer and 353 controls. We applied independent discovery (ColoCare Study: n = 180 patients/n = 153 controls) and replication (CORSA Study: n = 88 patients/n = 200 controls) sets using an identical metabolomics platform (Supporting Information Fig. S1).

The ColoCare Study, in Heidelberg initiated in 2010, is an ongoing, international, multicenter prospective study including women and men newly diagnosed with primary colorectal cancer. Patients are recruited at the University Hospital of Heidelberg and the National Center for Tumor Diseases in Heidelberg, Germany. Participants provided consent prior to tumor resection if they met the following inclusion criteria: newly diagnosed colorectal cancer (both colon (ICD‐10 C18) and rectal or recto‐sigmoidal cancer (ICD‐10 C19/C20)), any stage of the disease, 18+ years at the time of diagnosis, and German‐speaking. EDTA blood samples from colorectal cancer patients were collected prior to surgery. Control participants were enrolled in the PRAEVENT Study, a population‐based study subjected to similar protocols and procedures, conducted at the National Center for Tumor Diseases in Heidelberg, Germany. All participants consented to take part in our study and EDTA blood samples were collected during a visit at the National Center for Tumor Diseases at recruitment (usually the same day after the consent dialog and after signing the informed consent form).

In the ongoing CORSA Study participants are recruited in cooperation with the province‐wide screening project “Burgenland Prevention Trial of Colorectal Disease with Immunological Testing” (B‐PREDICT), since 2003. All inhabitants of the Austrian province Burgenland aged between 40 and 80 years are invited annually to participate in fecal occult blood testing. Positive fecal occult blood tested individuals are subsequently offered a complete colonoscopy, and EDTA blood samples are collected prior to examination. Additional colorectal cancer patients are recruited at the General Hospital of Vienna (Department of Surgery), and at three additional hospitals in Vienna. All colorectal cancer patients included in the CORSA Study are individuals with histologically confirmed, sporadic colorectal cancer. CORSA controls are individuals who received a complete colonoscopy within the B‐PREDICT screening but exhibited no pathological findings of disease.

All colorectal cancer samples selected for inclusion into the presented study were collected prior to any clinical treatment, including surgery or neo‐adjuvant therapy, and did not have a prior history of cancer. Controls included in the study can be considered as “cancer‐free”; having no prior history of cancer. Patients and controls were 95% of Caucasian origin, recruited within the last 15 years and selected to be matching according to their recruitment time point. Clinical data, including tumor location, staging, and treatment history were abstracted from medical records. Demographic characteristics (e.g. age, weight, height and smoking status) were assessed by study‐specific questionnaires. All clinical and demographic data were harmonized across all cohorts.

### Sample collection and analysis

In both cohorts, nonfasted EDTA blood samples were collected and processed within 4 h, according to identical processing protocols, and stored at −80 °C. Samples at each respective study site were shipped on dry ice to the International Agency for Research on Cancer (IARC) in Lyon, France for analysis. Samples were analyzed with a ultrahigh performance liquid chromatography‐ quadrupole time‐of‐flight mass spectrometry (UHPLC‐QTOF‐MS) system (Agilent Technologies) consisting of a 1,290 Binary LC system, a Jet Stream electrospray ionization (ESI) source, and a 6,550 QTOF mass spectrometer. Samples from each study center were analyzed in cohort‐specific batches, which consisted of five and six 96‐well plates for CORSA and ColoCare, respectively.

A detailed overview of the sample preparation and a complete description of sample analysis by UHPLC‐QTOF‐MS, pre‐processing of metabolomics data can be found in Supporting Information File S1. A summary of the data processing workflow is shown in Supporting Information Figure S1.

### Data analysis

Features with missing values in >50% of either colorectal cancer patient or control samples in both populations were excluded from analysis. The remaining maximum 50% of missing values were not imputed according to the recommendations of Di Guida *et al*. 21. Blank adjustment was applied for the ColoCare and CORSA samples separately; features that had a minimum relative mean intensity below the relative mean intensity of blank samples were removed. “Features” were defined as chromatographic peaks formed by specific ions, while “compounds” or “metabolites” referred to a confirmed molecule that can consist of one or more features (adducts, clusters and fragments).

Feature intensities were log transformed using the natural logarithm prior to statistical analysis, to prevent heteroscedasticity.^21^, ^22^ Demographic and clinical characteristics are presented as medians with the interquartile range (IQR), or as numbers with corresponding percentages. Body mass index (BMI) was calculated as weight (kg) divided by the square of height (m^2^). BMI status was categorized based on the recommendations from the World Health Organization (WHO): underweight (<18.5 kg/m^2^), normal weight (18.5–24.9 kg/m^2^), overweight (25.0–29.9 kg/m^2^) and obese (≥30.0 kg/m^2^). Smoking status was categorized as current, former, and never.

#### *Discovery stage*

The discovery analysis was conducted in ColoCare samples. Log standardized odds ratios (OR.std) and 95% confidence intervals (CIs) were calculated using multiple logistic regression models with disease state as dependent variable to test the association with feature intensities. The OR.std represents the change in colorectal cancer occurrence when there is a one standard deviation (SD) change in metabolite intensity, allowing comparison of effect sizes between different features. Since odds ratios were standardized, the SD of the controls were used to calculate the OR.std. Sex, age, BMI (continuous), and smoking status were included as covariates in the final model. Features that showed significant differences between colorectal cancer patients and controls after correction for multiple testing, using False Discovery Rate (FDR) correction, in the discovery stage were carried forward to the replication stage. *A priori,* an FDR *p*‐value <0.05 was considered statistically significant.

#### *Replication stage*

The replication stage was conducted in CORSA Study samples. Significant features (FDR *p* < 0.05) from the discovery stage were analyzed in the replication stage using the same modeling approach as in the discovery stage. Features were tested if they point in the same direction as the corresponding effects in the discovery stage (one‐sided testing). Analyses were checked for any influence by analytical batch, but no marked effect could be identified in both stages. Features with significant test results were selected for identification using authentic chemical standards at IARC. A detailed overview of metabolite identification is explained in Supporting Information Table S1. When more than one mass spectrometry feature corresponded with a metabolite, the feature with the highest intensity was selected and presented in the manuscript (Supporting Information Table S2).

Spearman correlation analysis was used to identify metabolite‐metabolite correlations among all identified metabolites and to understand the intra‐relation of metabolites. Spearman correlation coefficients were calculated for all pairs of annotated features for samples from the discovery and replication set to account for deviations from linearity. All statistical analyses were performed in R, version 3.3.3.^23^

## Results

### Participant characteristics

Characteristics of the study population are summarized in Table 1. The ColoCare cohort consisted of 63% men in the colorectal cancer group and 38% men in the control group. In addition, ColoCare controls had on average less participants classified as overweight compared to the colorectal cancer patients. The CORSA cohort consisted of 68% men in the colorectal cancer group and 65% men in the controls group. Control patients from the CORSA cohort had on average slightly more participants categorized as overweight compared to colorectal cancer patients.

**Table 1:** Demographics and clinical characteristics of colorectal cancer patients and controls^1^

		Discovery stage: ColoCare Study		Replication stage: CORSA	
		CRC patients	Controls	CRC patients	Controls
Number of participants		*180*	*153*	*88*	*200*
Sex* ‡	*Men* n(%)	114 (63.3)	59 (38.6)	60 (68.2)	130 (65.0)
Age* ¥ ‡	*Median (IQR)*	66.0 (58.0–73.0)	51.0 (42.0–63.0)	70.0 (60.0–76.0)	64.0 (57.0–74.0)
Body mass index^2^^,*,‡^ (kg/m^2^)	*Median (IQR)*	26.4 (24.1–29.2)	23.5 (22.0–26.7)	26.1 (23.8–29.4)	27.0 (24.9–30.1)
	*Underweight, <18.5 n(%)*	2 (1.1)	19 (12.4)	0 (0)	2 (1.0)
	*Normal weight, 18.5–24.9 n(%)*	59 (32.8)	3 (2.0)	26 (29.5)	47 (23.5)
	*Overweight, 25–29.9 n(%)*	82 (45.6)	91 (59.5)	38 (43.2)	94 (47.0)
	*Obese, ≥30 n(%)*	37 (20.6)	35 (22.9)	15 (17.0)	50 (25.0)
Smoking status* n(%)	*Current*	30 (16.6)	25 (16.3)	20 (22.7)	25 (12.5)
	*Former*	77 (42.8)	47 (30.7)	30 (34.2)	64 (32.0)
	*Never*	61 (33.9)	75 (49.1)	35 (39.7)	104 (52.0)
	*Unknown*	12 (6.7)	6 (3.9)	3 (3.4)	7 (3.5)
Stage^3^^,†^ n(%)	*0*	7 (3.9)	‐	0 (0)	‐
	*I*	34 (18.9)	‐	30 (34.1)	‐
	*II*	66 (36.7)	‐	17 (19.3)	‐
	*III*	47 (26.1)	‐	18 (20.5)	‐
	*IV*	25 (13.9)	‐	12 (13.6)	‐
	*Unspecified*	1 (0.5)	‐	3 (3.4)	‐
	*Unknown*	0 (0)	‐	8 (9.1)	‐
Tumor location^4^^,†^ n(%)	*Colon – distal*	53 (29.4)	‐	21 (31.8)	‐
	*Colon – proximal*	57 (31.7)	‐	33 (28.4)	‐
	*Rectum*	70 (38.9)	‐	34 (39.8)	‐

1 Controls are defined as individuals not diagnosed with any colorectal malignancy.

2 Missing BMI for n = 5, 9 and 7 for ColoCare controls, CORSA CRC patients and controls, respectively.

3 TNM for colorectal cancer with neo‐adjuvant therapy and colon cancers without surgery (28.7%), else pTNM was used (71.3%).

4 Distal colon: sigmoid colon, descending colon, splenic flexure; Proximal colon: transverse colon, hepatic flexure, ascending colon, cecum, appendix; Rectum: rectum, rectosigmoid junction.

¥ *p*‐value <0.05 between ColoCare and CORSA CRC.

† *p*‐value <0.05 between CRC.

‡ *p*‐value <0.05 between controls.

* *p*‐value <0.05 between ColoCare and CORSA controls.

In both cohorts control groups consisted of more participants categorized as never smokers than compared to the colorectal cancer patients. In general, the distributions of covariates were relatively comparable between the discovery and replication cohorts. Controls from the ColoCare cohort were 13 years younger than controls from the CORSA cohort. The majority of participants have a BMI classified as overweight, except for the controls from ColoCare.

### Metabolic profiles discriminating between colorectal cancer patients and controls

Metabolomics analysis yielded 10,015 mass spectrometry features, defined as a chromatographic peak formed by specific ions that were identified across all study samples. After data pre‐processing, 1,156 and 1,148 features were carried forward for ColoCare and CORSA samples, respectively.

Next, 691 out of 1,156 features were found to be statistically significantly associated with disease state (discovery stage) after FDR correction and adjustment for age, sex, BMI, and smoking status. The 691 significant features were subsequently analyzed in the replication dataset, i.e. the CORSA Study samples. Of these features, 97 differed between CORSA patients and controls.

**Figure 1:**
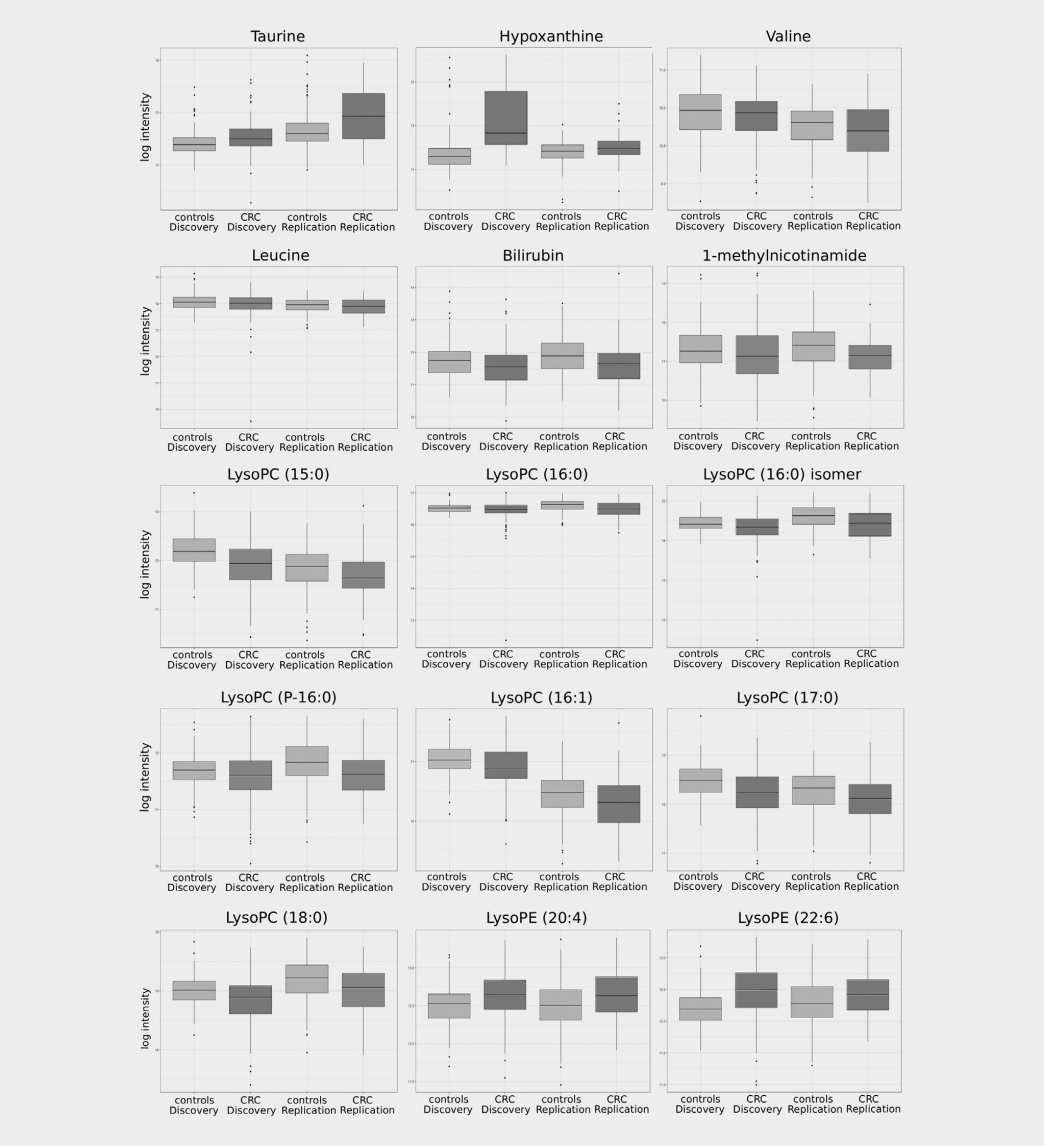
Box plots of the 15 annotated metabolites differentiating colorectal cancer patients (CRC) and controls for the discovery and replication set. The box plot presents the minimum, first quartile, median, third quartile and maximum log transformed relative intensity values and potential outliers of taurine, hypoxanthine, valine, leucine, bilirubin, 1‐methylnicotinamide (MNA), LysoPC (15:0), LysoPC (16:0), LysoPC (16:0) isomer, LysoPC (P‐16:0), LysoPC (16:1), LysoPC (17:0), LysoPC (18:0), LysoPE (20:4) and LysoPE (22:6), respectively.

The 97 replicated discriminating mass spectrometry features corresponded to 28 metabolites, defined as a confirmed molecule that can consist of one or more features (adducts, clusters and fragments) (Supporting Information Table S2). Six metabolites (taurine, hypoxanthine, valine, leucine, bilirubin, and 1‐methylnicotinamide) were identified using authentic standards resulting in a level 1 identification according to the Metabolomics Standards Initiative (MSI), nine compounds (seven lysophosphatidylcholines (LysoPCs) and two lysophosphatidylethanolamines (LysoPEs)) reached MSI level 2 identification, and 14 compounds could not be identified (unknown metabolites, MSI level 4). The intensity of these 15 identified metabolites exhibited significant differences between colorectal cancer patients and controls in both the discovery and replication set (Fig. 1). Taurine, hypoxanthine, LysoPE (20:4), and LysoPE (22:6) showed higher relative mean intensity values in the colorectal cancer group compared to controls, representing a OR.std higher than one (Table 2). Valine, leucine, bilirubin, 1‐methylnicotinamide, and seven LysoPCs (LysoPC(15:0), LysoPC (16:0), LysoPC(16:0) isomer, LysoPC(P‐16:0), LysoPC(16:1), LysoPC(17:0), LysoPC(18:0)) showed higher relative mean intensity values in the control group compared to the colorectal cancer patient group, indicating an OR.std lower than one (Table 3).

**Table 2:** List of replicated metabolites *positively* associated with colorectal cancer^1^

Compound Name	ID level^2^	Discovery stage: ColoCare Study				Replication stage: CORSA			
		*CRC* Mean ± SD^3^	*Controls* Mean ± SD^3^	*OR.std* 4 [95% CI]	*p* value^5^	*CRC* Mean ± SD^3^	*Controls*Mean ± SD^3^	*OR.std* 4 [95% CI]	*p* value^5^
*Taurine*	1	12.53 ± 0.28	12.41 ± 0.24	1.44 [1.13; 1.87]	7.2E‐03	12.96 ± 0.49	12.66 ± 0.34	1.95 [1.53; 2.51]	4.2E‐06
*Hypoxanthine*	1	13.18 ± 1.32	11.73 ± 0.91	3.07 [2.25; 4.45]	9.1E‐17	12.00 ± 0.53	11.79 ± 0.49	1.43 [1.09; 1.92]	3.6E‐02
*LysoPE(20:4)*	2	12.64 ± 0.30	12.50 ± 0.26	1.37 [1.08; 1.76]	2.0E‐02	12.66 ± 0.32	12.51 ± 0.32	1.51 [1.15; 2.01]	1.4E‐02
*LysoPE(22:6)*	2	12.48 ± 0.40	12.21 ± 0.29	1.80 [1.42; 2.31]	1.5E‐06	12.41 ± 0.34	12.28 ± 0.36	1.60 [1.20; 2.17]	7.8E‐03
*171.0292@2.8578448*	4	10.32 ± 0.52	10.04 ± 0.40	1.61 [1.26; 2.08]	3.4E‐04	10.2 ± 0.54	9.81 ± 0.41	2.04 [1.52; 2.81]	3.5E‐05
*279.1473@2.7668757*	4	11.08 ± 0.83	10.59 ± 0.66	1.38 [1.09; 1.77]	1.5E‐02	10.64 ± 0.84	9.97 ± 0.66	2.20 [1.62; 3.08]	1.0E‐05
*428.3615@7.4910417*	4	9.61 ± 0.34	9.49 ± 0.26	1.49 [1.07; 2.15]	3.3E‐02	9.56 ± 0.25	9.45 ± 0.18	1.59 [1.14; 2.26]	2.8E‐02

1 Controls are defined as individuals not diagnosed with any colorectal malignancy.

2 According to MSI.

3 Log transformed relative intensity values.

4 OR.std: standardized Odds Ratio, represents the relative change in colorectal cancer (CRC) risk when there is a one standard deviation (SD) change in metabolite intensity. OR.std is based on the SD of the controls.

5 *p*‐value: FDR‐corrected *p*‐value.

**Table 3:** List of replicated metabolites *inversely* associated with colorectal cancer^1^

Compound Name	ID level^2^	Discovery stage: ColoCare Study				Replication stage: CORSA			
		CRCMean ± SD^3^	Controls Mean ± SD^3^	OR.std^4^ [95% CI]	*p* value^5^	CRC Mean ± SD^3^	ControlsMean ± SD^3^	OR.std^4^ [95% CI]	*p* value^5^
*Leucine*	1	13.98 ± 0.49	14.07 ± 0.31	**0.69** [0.53; 0.87]	4.4E‐03	13.86 ± 0.32	13.93 ± 0.28	**0.72** [0.55; 0.93]	4.7E‐02
*1‐methylnicotinamide*	1	11.18 ± 0.68	11.33 ± 0.60	**0.69** [0.53; 0.88]	6.5E‐03	11.11 ± 0.45	11.36 ± 0.56	**0.62** [0.46; 0.83]	6.8E‐03
*Valine*	1	10.36 ± 0.33	10.43 ± 0.33	**0.74** [0.56; 0.96]	4.2E‐02	10.18 ± 0.35	10.27 ± 0.27	**0.69** [0.53; 0.89]	1.7E‐02
*Bilirubin*	1	11.54 ± 0.59	11.74 ± 0.56	**0.57** [0.43; 0.75]	1.5E‐04	11.66 ± 0.64	11.92 ± 0.62	**0.66** [0.49; 0.87]	1.5E‐02
*LysoPC(16:1)*	2	10.90 ± 0.35	11.02 ± 0.27	**0.60** [0.46; 0.77]	1.2E‐04	10.31 ± 0.46	10.44 ± 0.36	**0.73** [0.57; 0.93]	4.4E‐02
*LysoPC(P‐16:0)*	2	11.58 ± 0.42	11.68 ± 0.28	**0.76** [0.61; 0.94]	2.0E‐02	11.63 ± 0.42	11.81 ± 0.39	**0.66** [0.50; 0.87]	1.4E‐02
*LysoPC(15:0)*	2	11.90 ± 0.49	12.19 ± 0.36	**0.53** [0.40; 0.67]	2.8E‐07	11.69 ± 0.48	11.83 ± 0.43	**0.71** [0.55; 0.92]	4.2E‐02
*LysoPC (16:0)*	2	16.44 ± 0.38	16.52 ± 0.13	**0.75** [0.61; 0.91]	5.7E‐03	16.49 ± 0.26	16.61 ± 0.19	**0.59** [0.46; 0.76]	3.3E‐04
*LysoPC(16:0) isomer*	2	14.28 ± 0.45	14.44 ± 0.21	**0.71** [0.55; 0.90]	8.3E‐03	14.42 ± 0.38	14.59 ± 0.30	**0.58** [0.42; 0.78]	2.9E‐03
*LysoPC(17:0)*	2	12.22 ± 0.47	12.47 ± 0.36	**0.54** [0.41; 0.69]	8.9E‐07	12.11 ± 0.47	12.27 ± 0.40	**0.68** [0.52; 0.88]	1.7E‐02
*LysoPC(18:0)*	2	14.84 ± 0.39	15.00 ± 0.25	**0.63** [0.50; 0.78]	5.7E‐05	15.00 ± 0.42	15.18 ± 0.34	**0.63** [0.49; 0.81]	2.9E‐03
*157.1107@0.8299292*	4	11.45 ± 0.65	11.44 ± 0.61	**0.69** [0.52; 0.90]	1.3E‐02	11.39 ± 0.68	11.52 ± 0.66	**0.67** [0.49; 0.90]	3.2E‐02
*181.1113@5.475981*	4	10.37 ± 0.51	10.75 ± 0.47	**0.41** [0.30; 0.55]	5.4E‐09	10.88 ± 0.42	10.95 ± 0.33	**0.72** [0.55; 0.93]	4.2E‐02
*190.0061@0.83498704*	4	10.93 ± 0.32	11.04 ± 0.24	**0.61** [0.46; 0.79]	3.3E‐04	11.07 ± 0.39	11.21 ± 0.32	**0.66** [0.51; 0.84]	6.1E‐03
*247.0286@3.3104632*	4	12.88 ± 0.43	13.14 ± 0.31	**0.43** [0.31; 0.57]	2.6E‐09	12.99 ± 0.40	13.13 ± 0.32	**0.66** [0.50; 0.86]	1.2E‐02
*445.8809@0.56495804*	4	13.86 ± 0.36	13.96 ± 0.11	**0.76** [0.64; 0.90]	3.0E‐03	13.79 ± 0.31	13.87 ± 0.16	**0.77** [0.61; 0.94]	4.2E‐02
*452.8025@7.3067145*	4	9.77 ± 0.26	9.85 ± 0.28	**0.72** [0.54; 0.96]	4.4E‐02	9.88 ± 0.23	9.97 ± 0.21	**0.65** [0.50; 0.86]	1.1E‐02
*535.2997@7.0497603*	4	11.01 ± 0.41	11.16 ± 0.35	**0.65** [0.49; 0.85]	3.4E‐03	11.27 ± 0.38	11.37 ± 0.33	**0.72** [0.55; 0.93]	4.4E‐02
*545.3467@7.2500143*	4	13.39 ± 0.53	13.50 ± 0.31	**0.79** [0.63; 0.98]	5.0E‐02	13.78 ± 0.46	14.00 ± 0.39	**0.61** [0.47; 0.78]	1.1E‐03
*610.9343@7.191048*	4	11.78 ± 0.43	11.94 ± 0.34	**0.63** [0.49; 0.80]	4.6E‐04	11.34 ± 0.40	11.51 ± 0.33	**0.63** [0.48; 0.82]	3.9E‐03
*638.9663@7.4319897*	4	10.85 ± 0.33	10.97 ± 0.28	**0.68** [0.53; 0.87]	4.7E‐03	10.98 ± 0.29	11.13 ± 0.24	**0.58** [0.44; 0.76]	4.5E‐04

1 Controls are defined as individuals not diagnosed with any colorectal malignancy.

2 According to MSI.

3 Log transformed relative intensity values.

4 OR.std: standardized Odds Ratio, represents the relative change in colorectal cancer (CRC) risk when there is a one standard deviation (SD) change in metabolite intensity. OR.std is based on the SD of the controls.

5 *p*‐value: FDR‐corrected *p*‐value.

### Correlation analysis

**Figure 2:**
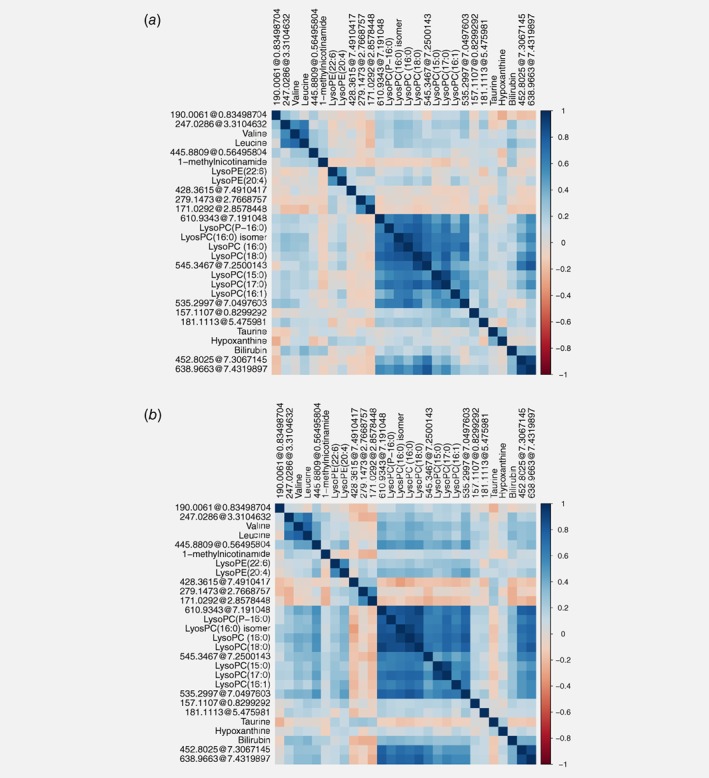
Metabolite‐metabolite correlation analysis of replicated metabolites. Positive correlations are highlighted in blue, negative correlations are highlighted in red. Unknown compounds are indicated as monoisotopic mass@retention time. Metabolites are ordered by hierarchical clustering. (*a*) Spearman correlation analysis plot of the discovery dataset. (*b*) Spearman correlation analysis plot of the replication dataset.

Spearman correlation analysis was used to identify potential metabolite‐metabolite correlations among all identified metabolites (Fig. 2). Correlation patterns demonstrated similar results across the discovery (Fig. 2 *a*) and replication stage (Fig. 2 *b* **).** For both stages, all LysoPCs were positively correlated (Spearman correlation coefficient range [r_s_]: 0.40–0.91) but showed only a weak correlation to LysoPE (22:6) and LysoPE (20:4). Valine and leucine were highly correlated (discovery stage r_s_: 0.73, replication stage r_s_: 0.78). In addition, the majority of replicated compounds annotated as unknown (n = 13) were correlated with each other but showed only weak correlations with the other annotated compounds. Spearman correlation coefficients are shown in Supporting Information Table S3.

## Discussion

In our study, we identified plasma metabolites that are associated with colorectal cancer and which were replicated in an independent study population. We found 28 metabolites associated with disease state in two independent study cohorts, the ColoCare and CORSA studies. In total, 15 out of 28 metabolites could be identified. Taurine, hypoxanthine, valine, leucine, LysoPCs, and LysoPEs have been reported to be linked with colorectal cancer in previous metabolomics studies. All LysoPCs were positively correlated, valine and leucine were highly correlated, and the majority of unidentified metabolites were correlated with each other. Except for valine and leucine, the identified metabolites were only slightly or not correlated with each other.

Taurine was previously shown to be increased in serum of 60 colorectal cancer patients compared to 60 apparently healthy individuals 13 and in tumor tissue of 16 colorectal cancer patients; 24 which is in agreement with our findings. Recent studies have suggested taurine as a microbiota‐associated metabolite playing a mediating role in microbiome‐host interactions.^25^, ^26^ Given the knowledge that gut microbiota differ between colorectal cancer patients and healthy individuals, and that microbial composition is linked to colorectal cancer risk,^27^ taurine presents a promising candidate for further investigation.

Hypoxanthine has been previously reported to be increased in tumor tissue of colorectal cancer patients compared to normal tissue of healthy individuals.^15^ In contrast, a recent study, published by Long *et al*. reported decreased levels of hypoxanthine in colorectal cancer and polyps compared to controls.^20^ Like taurine,^5^ hypoxanthine is an antioxidant and increased levels reported in our study may be the result of increased oxidative stress,^28^ which is recognized as an important process in carcinogenesis, including colorectal cancer.^29^, ^30^ Inconsistent findings in hypoxanthine levels may be due to the type of specimen analyzed, or lack of statistical power because of lower sample numbers included. Furthermore, a possible reason for the inconsistent hypoxanthine levels may be caused by red blood cell hemolysis during the preparation of serum samples utilized in the Long study in contrast to plasma used in the present analysis.^31^

With respect to branched‐chain amino acids (BCAAs), we observed that valine was reduced among colorectal cancer patients compared to controls. This result is consistent with two prior studies; Ma and colleagues compared serum of 30 colorectal cancer patients to 30 colorectal cancer‐free controls,^12^ and Farshidfar *et al*. investigated metabolomic signatures in colorectal cancer serum of stage I‐IV patients.^14^ Comparable to valine, decreased plasma levels of leucine were also reported in our colorectal cancer patients compared to controls. Decreased blood levels of BCAAs could reflect increased requirement for amino acids due to the high protein turnover in the malignant setting.^15^, ^19^, ^32^

Moreover, seven LysoPCs were detected at lower levels among colorectal cancer patients compared to controls. LysoPC (16:0) and LysoPC (18:0) were reported before to be lower in the plasma of colorectal cancer patients *versus* control individuals.^33^, ^34^ There seems to be a general trend of lower levels of LysoPCs among colorectal cancer patients in existing studies,^17^, ^33^, ^35^ which is in line with the findings reported in our study. This pattern might reflect an increased degradation rate of LysoPCs as a result of the accelerated cell proliferation rate of cancerous cells.^36^ It has been suggested that decreased levels of LysoPCs could result from weight loss and possibly inflammatory processes related to cancer.^37^, ^38^ While the majority of our study participants were classified as overweight, we did not have data on changes in body weight among patients prior to a colorectal cancer diagnosis.

LysoPE (20:4) and LysoPE (22:6) were increased in colorectal cancer patients compared to controls. LysoPEs belong to the group of signaling lipids and are constituents of cell membranes. Recently, serum LysoPEs were found to be elevated among breast cancer patients.^39^ However, knowledge is limited regarding the role of LysoPEs in healthy and diseased individuals.

We also identified a notable decrease in MNA, an inactive metabolite of nicotinamide,^40^ among colorectal cancer patients compared to controls. MNA has been reported *in vivo* to be involved in the COX‐2/PGI_2_ pathway,^40^ which plays a major role in inflammation and colorectal carcinogenesis.^41^, ^42^ In addition, this is the first metabolomics study to report lower plasma bilirubin levels in colorectal cancer patients compared to controls. Previously, a European study analyzing genomic alterations in promoter variants involved in bilirubin homeostasis, and another study investigating serum bilirubin levels in a large U.S. population have proposed a protective effect of bilirubin against colorectal carcinogenesis; 43, 44 our metabolomics findings carefully support this hypothesis. The underlying mechanisms of the relationship between bilirubin and colorectal cancer remain unclear.

Untargeted metabolomics is an elegant approach for the discovery of metabolites associated with cancer. However, one may wonder whether the seemingly small differences between colorectal cancer patients and controls are biologically relevant. It is important to keep in mind that findings presented are log transformed relative values. As a consequence, reported results hint towards the direction of the association and quantification of the metabolites is needed to be able to interpret absolute differences. Our results for taurine, hypoxanthine, valine, leucine, bilirubin, and 1‐methylnicotinamide suggest future research to investigate the underlying biological mechanism of these metabolites in relation to colorectal cancer.

A strength of the present study is the use of a discovery‐replication design leveraging two independent, relatively large, patient cohorts, both including patients of Caucasian origin, from two different countries. In general, untargeted methods typically yield data with high amounts of noise and nonbiological information.^45^ This makes replication of untargeted metabolomics findings within ethnically homogenous cohorts extremely valuable, as it enables the exclusion of features that are not robustly associated with the case–control status.

A limitation of our study is that due to recruitment procedures we tend to have more early stage colorectal cancer cases (stage I‐II) compared to advanced metastatic patients (stage IV). This may indicate that our findings are mostly associated with early metabolic changes in colorectal carcinogenesis rather than with metastatic formation. Furthermore, findings are derived from cross‐sectional data. Therefore, it is not possible to explore to which extent metabolites are causally related to cancer or cancer‐related changes. Lastly, although our study was performed using a single stringent metabolomics approach across two independent populations, we acknowledge that metabolomics assays can be conducted using a variety of analytical platforms. As such, future studies should include multiple platforms to ensure the highest analytical coverage of the metabolome. Technical progress and the development of more comprehensive metabolite databases will also be needed to improve annotation of unknown compounds, including the unknown metabolites in our study. Future targeted approaches, allowing the quantitative measurement of metabolites, would allow quantification of their absolute concentrations.^46^, ^47^

In summary, our study provides new evidence of associations of colorectal cancer with plasma metabolites and also confirms some evidence of previous findings.

The combination of an untargeted metabolomics approach, a rigorous discovery‐replication design utilizing large sample sizes from independent cohorts, led to the identification and replication of 28 metabolites associated with colorectal cancer, including 15 metabolites that could be identified. These 15 identifiable metabolites should be carried forward as candidates for targeted analysis in prospective cohort studies, preferably derived from a colorectal cancer screening program, to verify their discriminating or potential predicting properties. Our study provides important leads for further studies focusing on metabolic differences between colorectal cancer‐free individuals, and patients with different stages of colorectal cancer. Together, our findings emphasize the power of metabolomics as a strong molecular approach for gaining novel insights regarding metabolic changes associated with colorectal cancer.

## Ethical approval and consent to participate

Written informed consent was obtained from all participants. The ColoCare Study has been approved by the ethics committee of the Medical Faculty at the University of Heidelberg. The CORSA Study was approved by the ethical review committee of the Medical University of Vienna (1160/2016), by the “Ethikkommission der Stadt Wien” (06‐150‐VK) and by the institutional review board “Ethikkommission Burgenland”. The study was also approved by the International Agency for Research on Cancer ethics committee.

## Disclosure

Michael M. Bergman is founder of the biotechnology company Vacthera BioTech GmbH (Vienna, Austria) and received a consultant fee (<2,000€) from Dr. Falk GmbH (Vienna, Austria). Cornelia M. Ulrich (Director of the Comprehensive Cancer Center at Huntsman Cancer Institute, Salt Lake City, USA) oversees research funded by several pharmaceutical companies but has not received funding directly herself.

## Supporting information


**Supplementary Figure S1 Workflow and study design.** (A) The study population consisted of 268 colorectal cancer patients and 353 controls (individuals not diagnosed with any colorectal malignancy), including an independent discovery (ColoCare Study: n = 180 patients/n = 153 controls) and replication set (CORSA Study: n = 88 patients/n = 200 controls). (B) Study workflow including feature finding and data alignment (raw data processing), data processing, data analysis (discovery‐replication setting) and identification of replicated features.

**Supplementary Table S1** Identification of metabolites

**Supplementary Table S2** Metabolite identifications of the 97 replicated features

**Supplementary Table S3** Spearman correlation coefficients of the 28 metabolites in the discovery and replication stage

**Appendix S1**: Supporting Information.
