# Supplementary material for: Plasma metabolites associated with colorectal cancer: A discovery‐replication strategy
Source: Int J Cancer. 2019 Feb 14;145(5):1221–31. doi: 10.1002/ijc.32146 (PMC6614008; doi:10.1002/ijc.32146)

Identification of metabolites

General notes:

- Chromatograms and spectra are from representative study samples and pure chemical standards when available. All chemical standards were from IROA Technologies' Mass Spectrometry Metabolite Library (MSMLS)
- Chromatograms and isotope patterns: Find Compounds by Formula searches in Agilent MassHunter Qualitative Analysis B.06.00 SP1
- Isotope patterns: Red rectangles represent an isotope pattern calculated from the elemental composition (shown in the title of each spectra) , bars inside the rectangles show the observed isotope peaks
- MS/MS spectra: Precursor is indicated with a blue dot above the ion. Collision energy is on top of the spectra, e.g. CID@20.0.

Table of contents

Page 1: 1-Methylnicotinamide, Taurine

Page 2: Valine

Page 3: Hypoxanthine, Leucine

Page 4: LysoPC(16:1)

Page 5: LysoPC(15:0), LysoPE(22:6)

Page 6: LysoPE(20:4), LysoPC(16:0) isomer

Page 7: LysoPC(16:0), LysoPC(P-16:0)

Page 8: LysoPC(17:0), LysoPC(18:0)

Page 9: Bilirubin

1-Methylnicotinamide

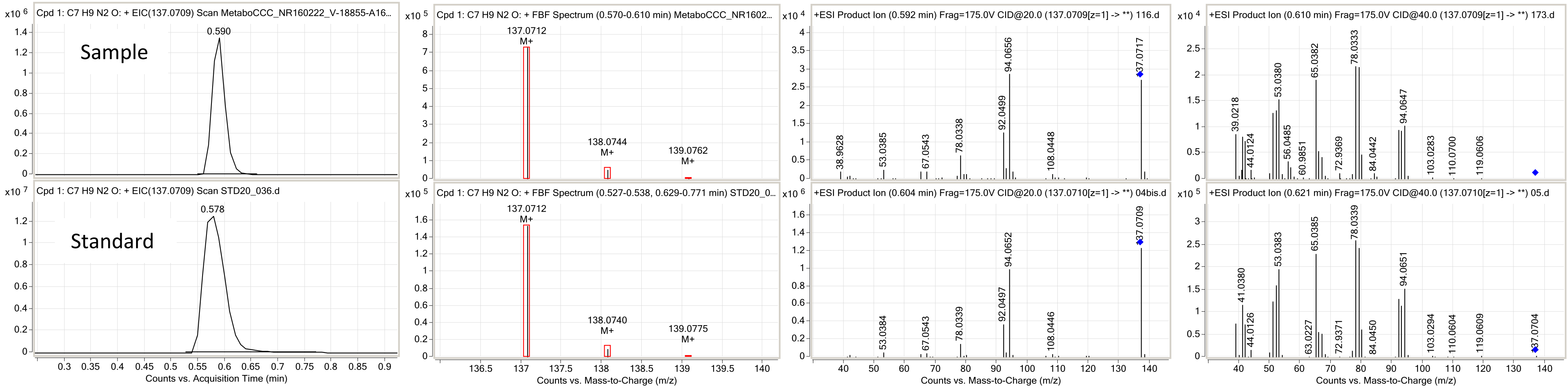

Taurine

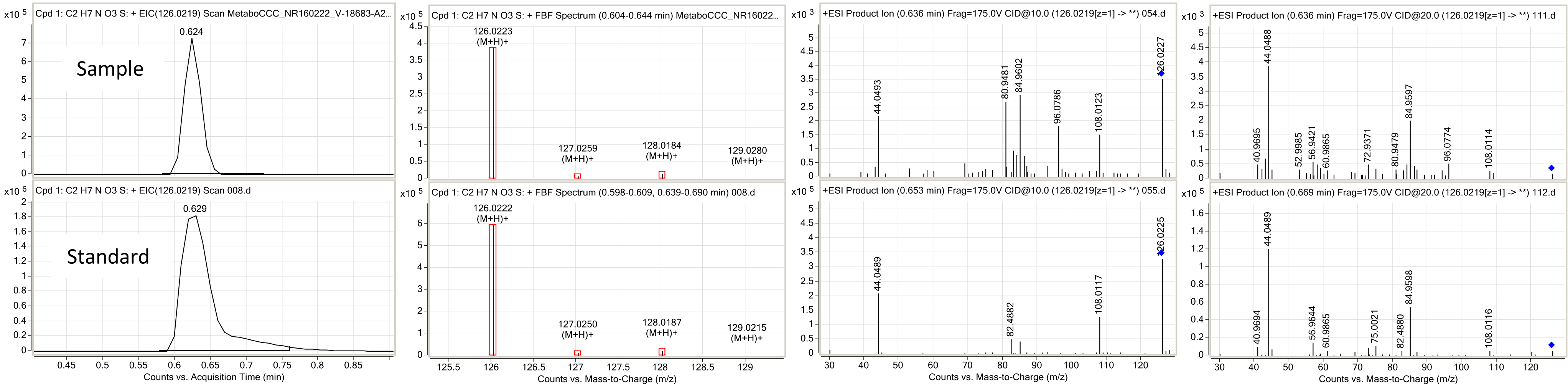

Valine

Valine was found as $[M+Na]^+$ due to saturation of the principal ion $[M+H]^+$. MS/MS spectra acquired from $[M+H]^+$

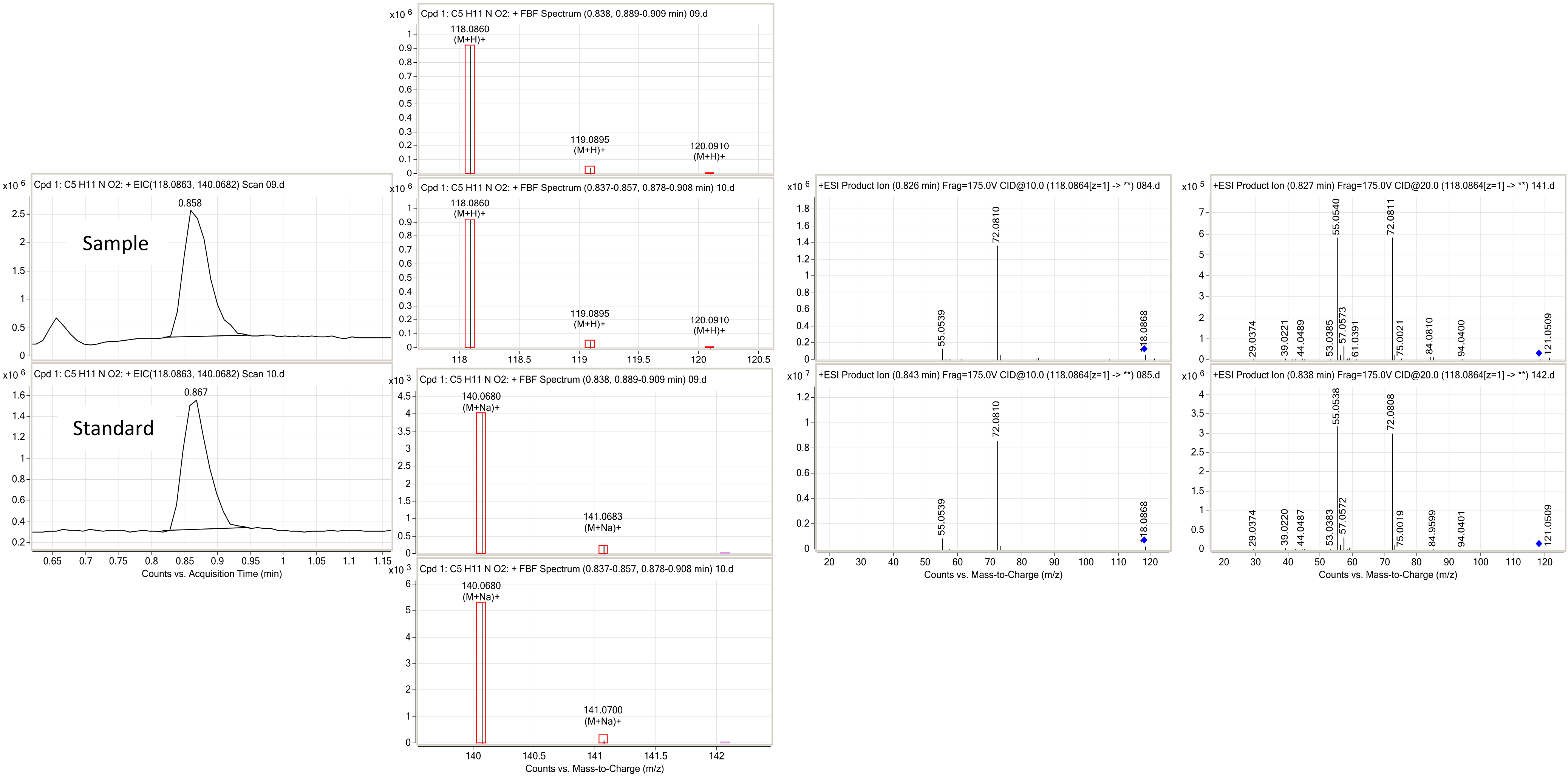

Hypoxanthine

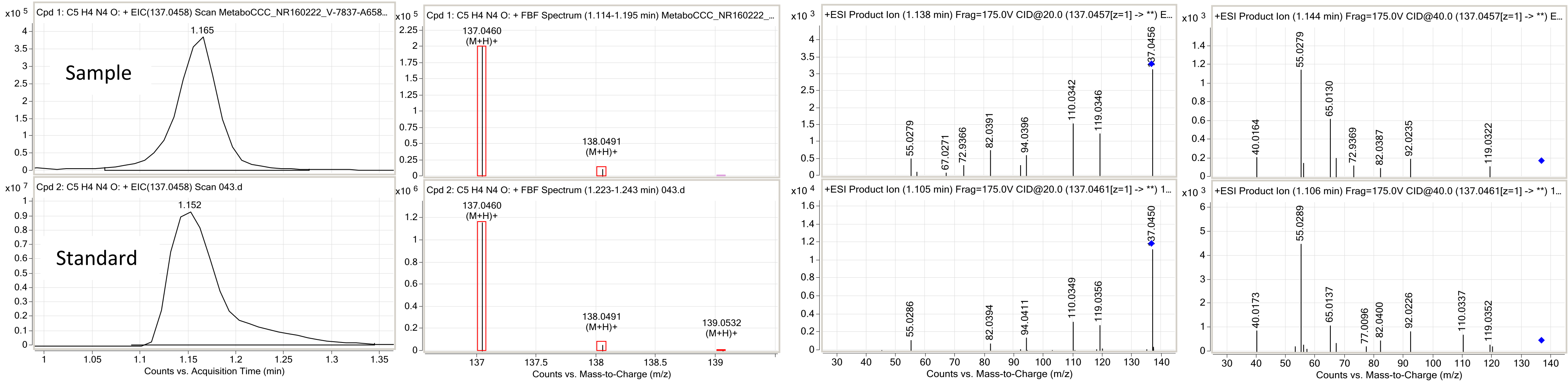

Leucine

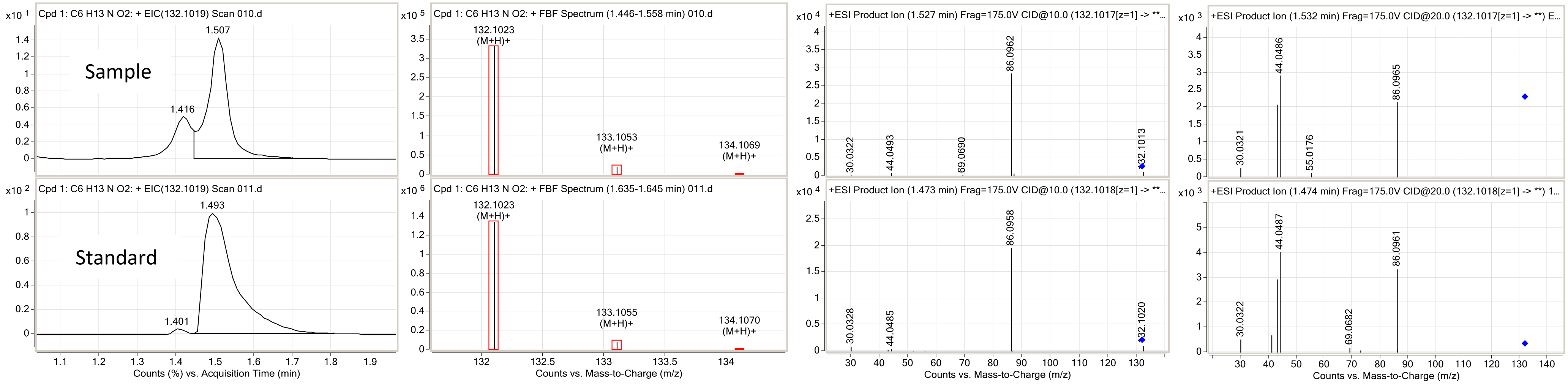

LysoPC(16:1)

LysoPC(16:1) found as $[M+H+K]^{2+}$. MS/MS was acquired from $[M+H]^{+}$

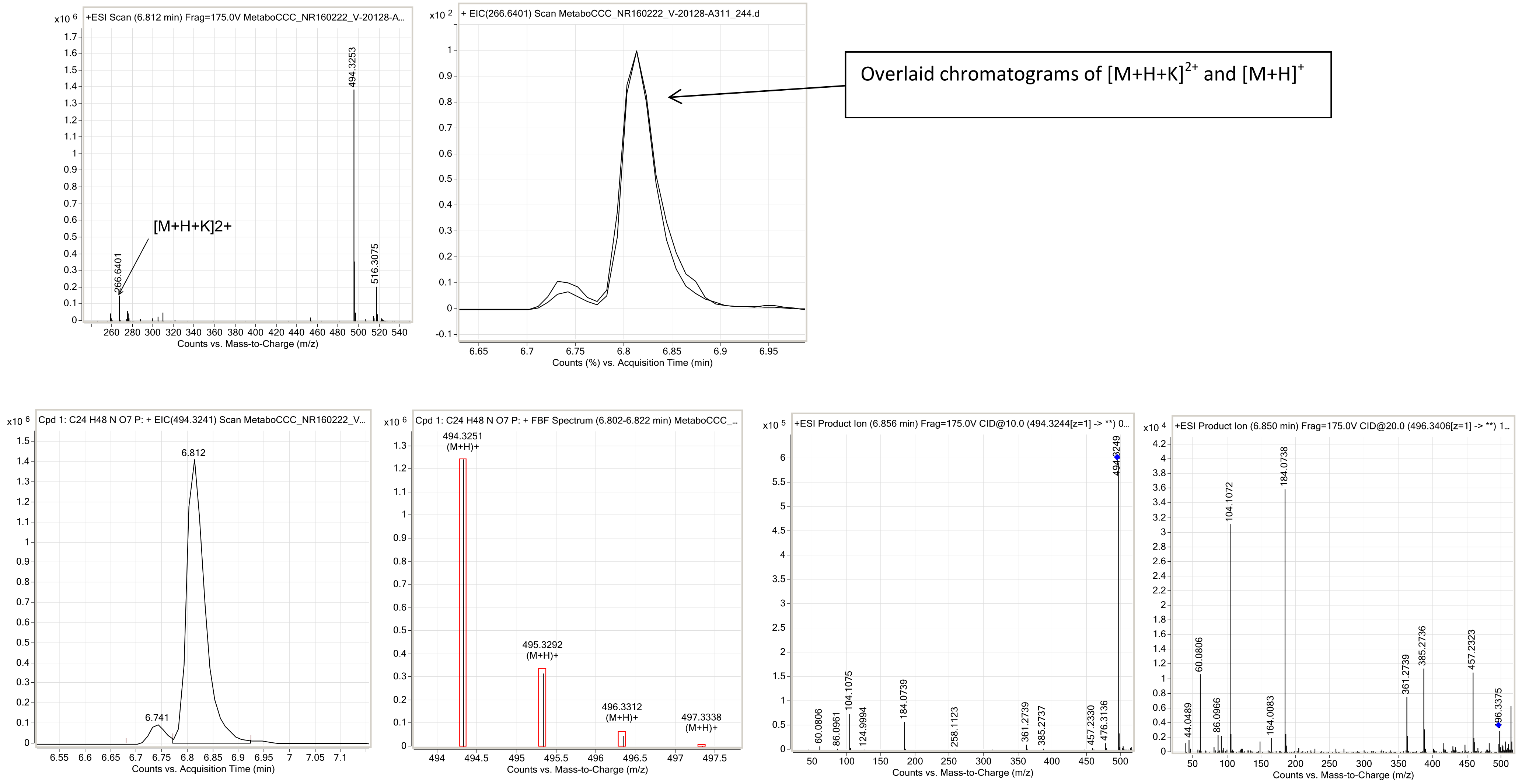

LysoPC(15:0)

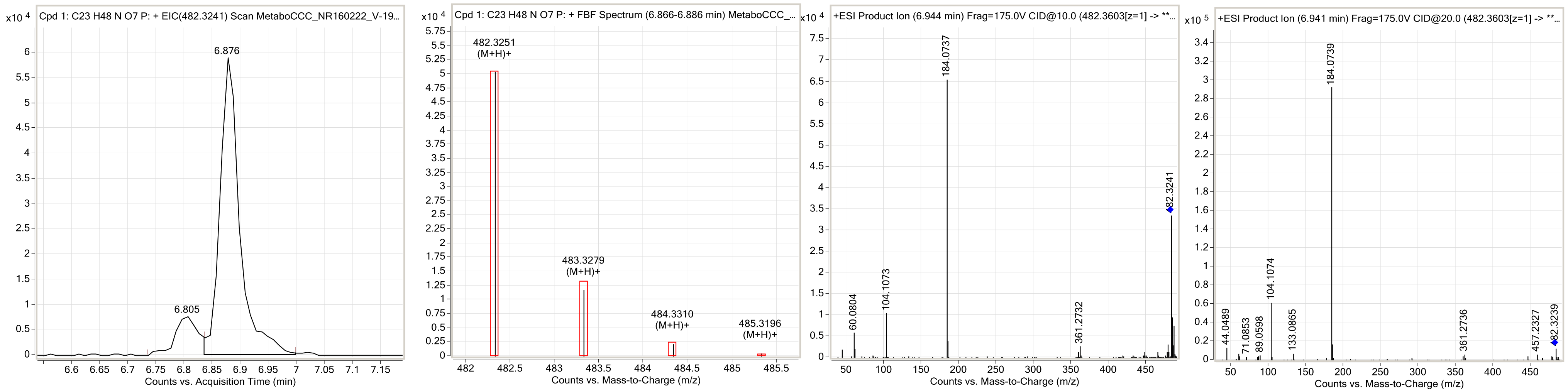

LysoPE(22:6)

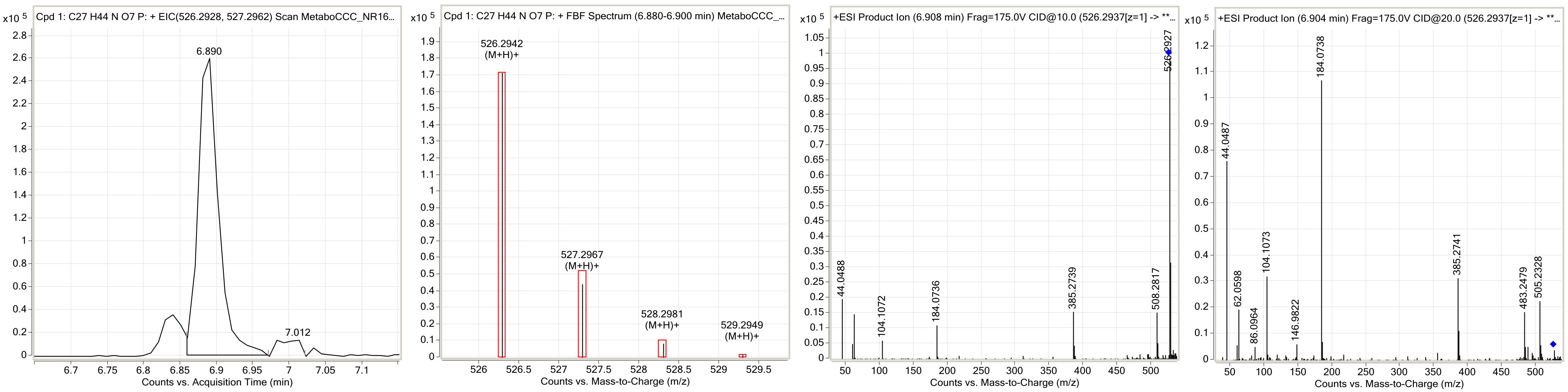

LysoPE(20:4)

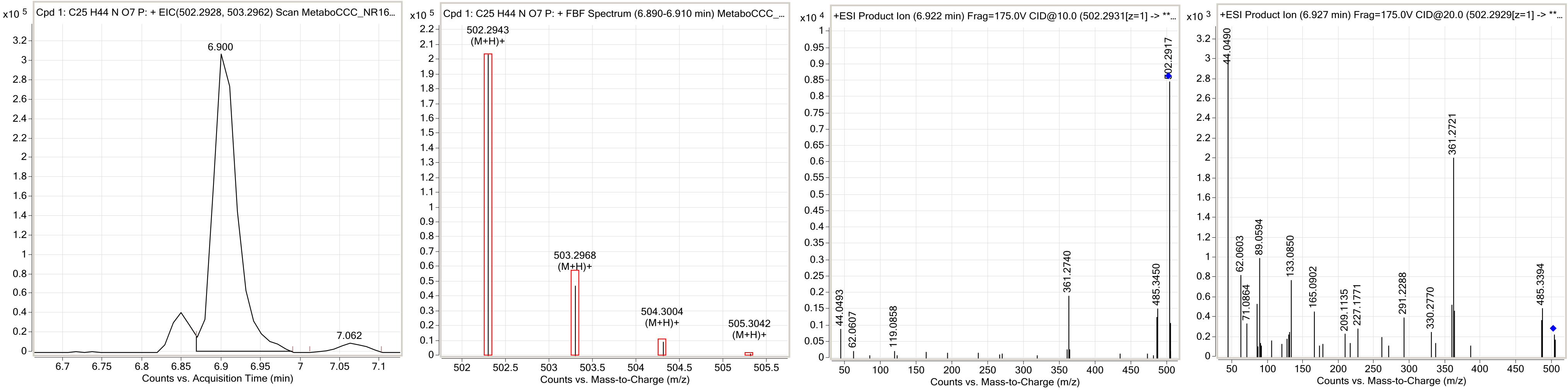

LysoPC(16:0) isomer

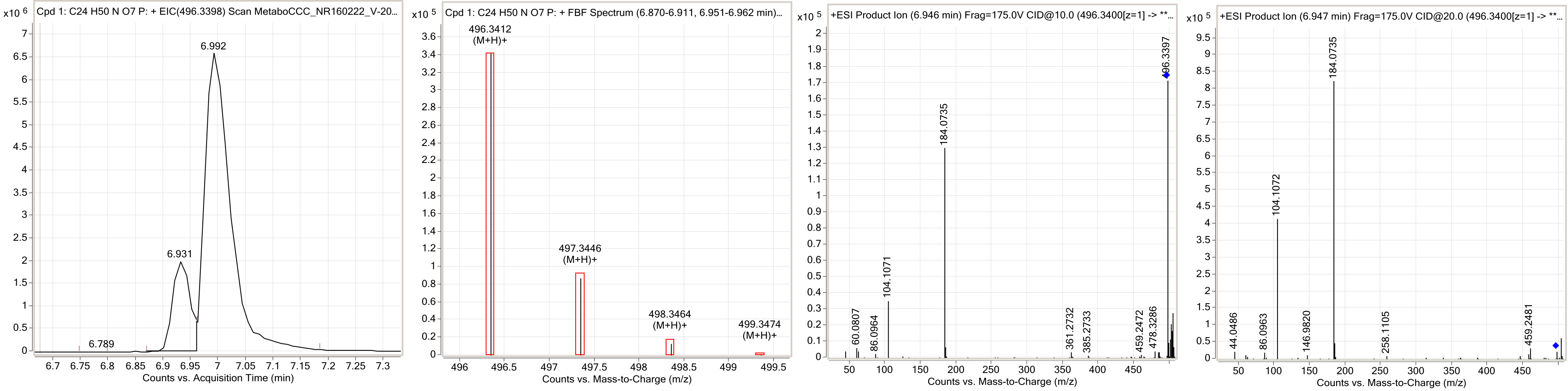

LysoPC(16:0)

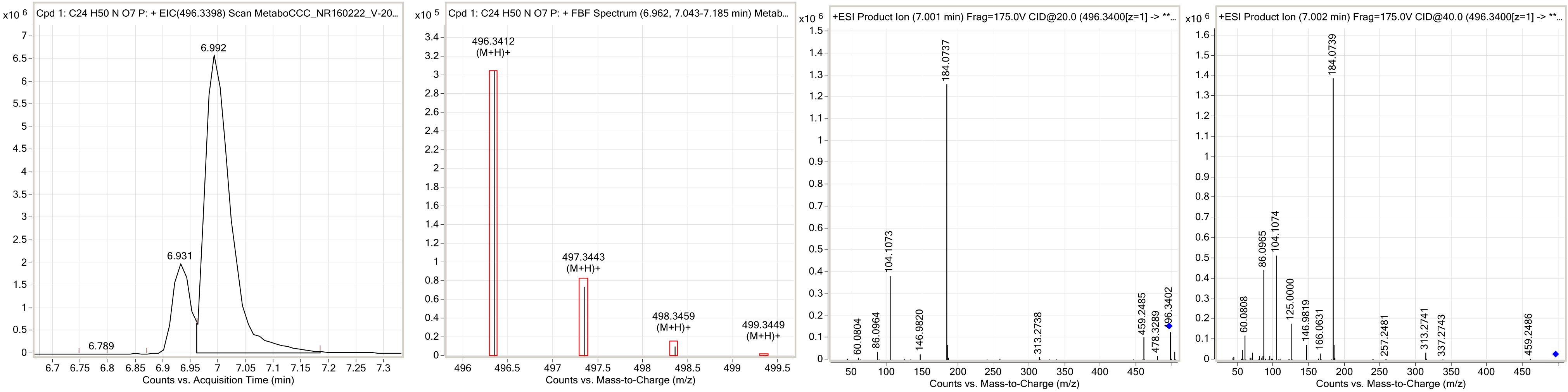

LysoPC(P-16:0)

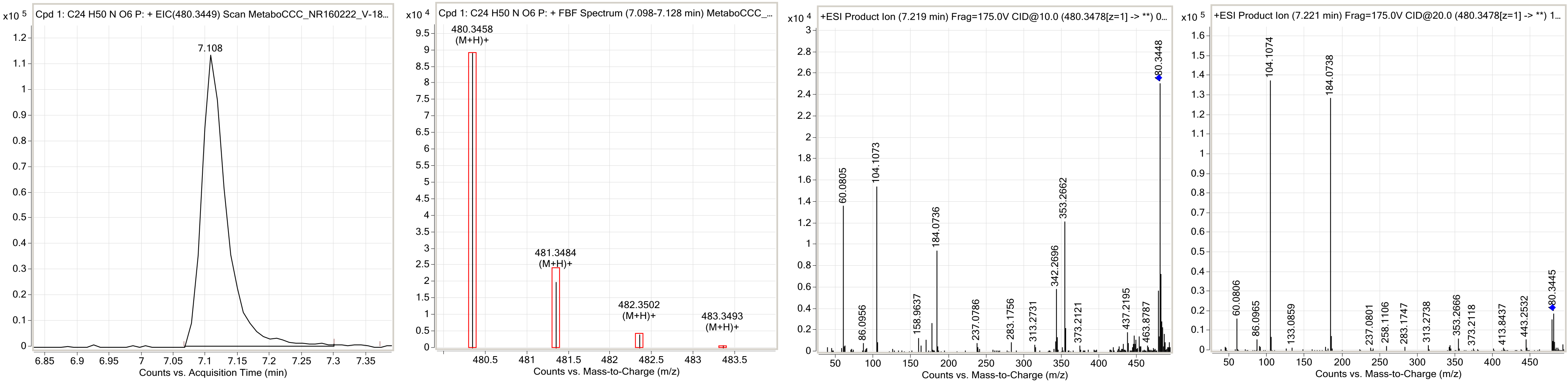

LysoPC(17:0)

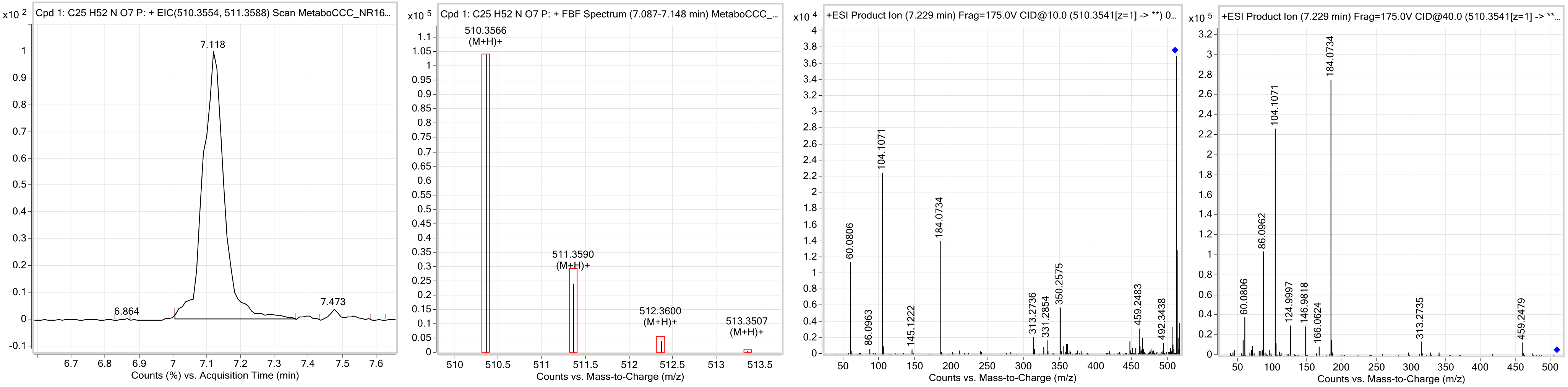

LysoPC(18:0)

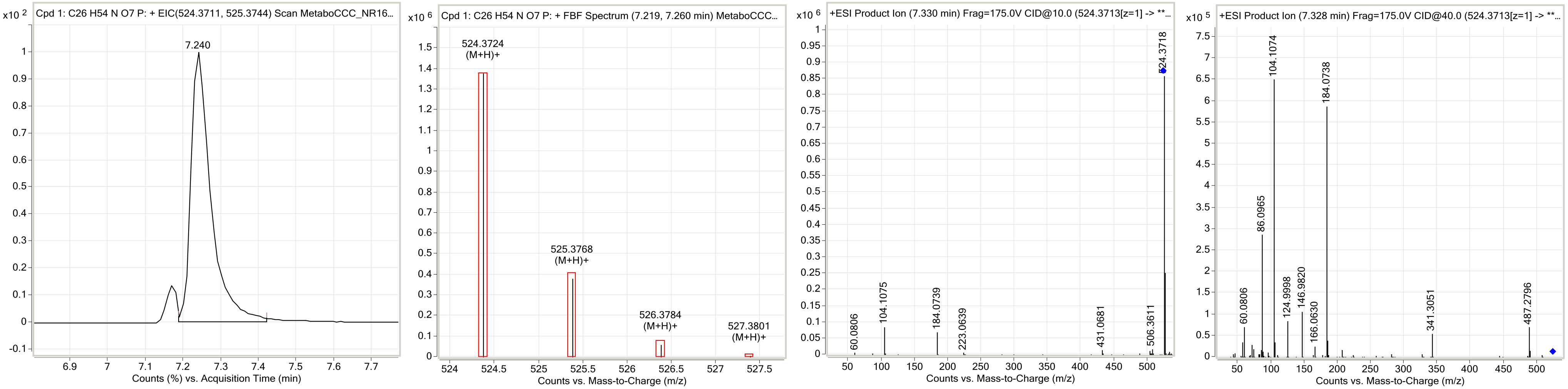

Bilirubin

Bilirubin typically exhibits high number of ions in electrospray (see isotope patterns below). MS/MS was acquired from $[M+H]^+$

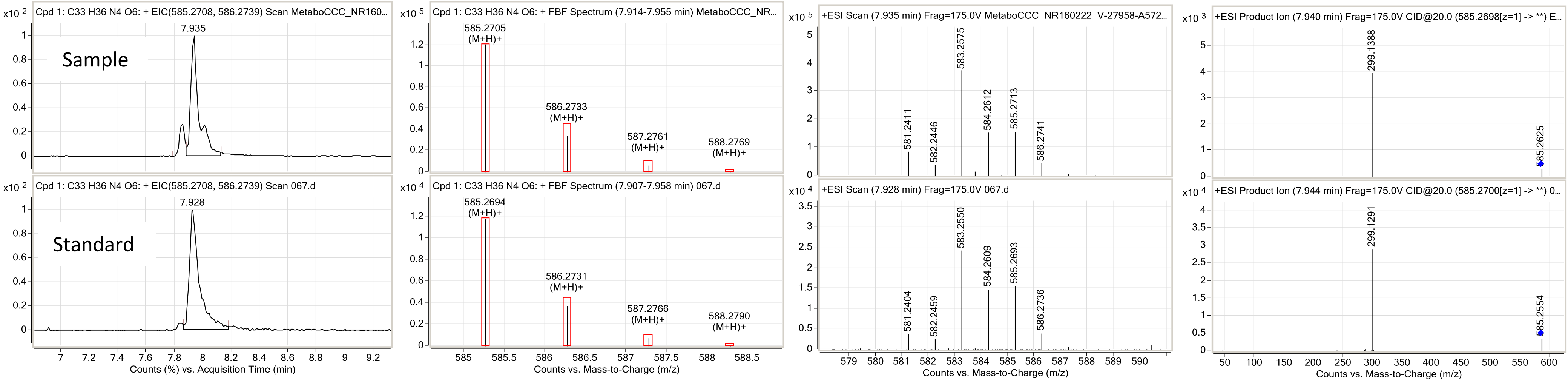

Supplement: Supplementary file 1 — Supplementary Figure S1 Workflow and study design. (A) The study population consisted of 268 colorectal cancer patients and 353 controls (individuals not diagnosed with any colorectal malignancy), including an independent discovery (ColoCare Study: n = 180 patients/n = 153 controls) and replication set (CORSA Study: n = 88 patients/n = 200 controls). (B) Study workflow including feature finding and data alignment (raw data processing), data processing, data analysis (discovery‐replication setting) and identification of replicated features. [file IJC-145-1221-s001.pdf]
