# Supplementary material for: Plasma metabolites associated with colorectal cancer: A discovery‐replication strategy
Source: Int J Cancer. 2019 Feb 14;145(5):1221–31. doi: 10.1002/ijc.32146 (PMC6614008; doi:10.1002/ijc.32146)
Supplement: Supplementary file 2 — Supplementary Table S1 Identification of metabolites [file IJC-145-1221-s002.pdf]

Supplementary Table S2. Metabolite identifications of the 97 replicated features

The red features represent the most intense feature belonging to a compound.

cpd#	monoisotopic mass@retention time	Compound name	Level according to the Metabolomics Standards Initiative
1	122.9206@0.56447005	Unknown	4
	205.8796@0.5669058		
	207.8776@0.56648636		
	273.8663@0.56767327		
	289.8411@0.5683634		
	341.8549@0.56762207		
	357.8274@0.56829727		
	409.8426@0.56771487		
	425.8154@0.5682527		
	445.8809@0.56495804		
	493.8012@0.56823456		
	513.8702@0.5653107		
	561.7904@0.5682394		
	565.8809@0.56176305		
	581.8576@0.56466657		
	623.8351@0.5579561		
	629.7794@0.56837577		
	649.8467@0.5632472		
	657.8285@0.55736333		
	691.8246@0.556467		
	697.7639@0.56842214		
	717.8281@0.5610313		
	765.7508@0.56839013		
	785.8171@0.5577929		
	853.8052@0.5547323		
	921.7903@0.55131626		
	937.7639@0.559643		
2	136.0636@0.58916837	**1-methylnicotinamide**	1
3	125.0146@0.6277308	**Taurine**	1
4	71.074@0.8027233	**Valine**	1

	139.0611@0.80280536		
5	157.1107@0.8299292	Unknown	4
6	190.0061@0.83498704	Unknown	4
7	136.0381@1.1621803	**Hypoxanthine**	1
7	*136.0384@0.85686004*		
8	85.0889@1.4623561	**Leucine**	1
9	279.1473@2.7668757	Unknown	4
10	171.0292@2.8578448	Unknown	4
11	143.0166@3.3154876	Unknown	4
	200.0362@3.3102927		
	214.0152@3.31054		
	231.9886@3.3105862		
	232.0561@3.3086596		
	232.0574@3.310062		
	245.0312@3.3104632		
	246.0422@3.310548		
	247.0286@3.3104632		
	250.0351@3.310955		
	261.0059@3.3104744		
	264.0146@3.3119278		
	282.0348@3.3101916		
12	181.1113@5.475981	Unknown	4
13	266.1351@6.8201194	**LysoPC(16:1)**	2
14	481.3159@6.8777747	**LysoPC(15:0)**	2
15	525.2864@6.888085	**LysoPE(22:6)**	2

16	501.285@6.8980646	**LysoPE(20:4)**	2
	523.2694@6.8969865		
17	495.3333@6.9326496	**LyosPC(16:0) isomer**	2
18	523.2949@6.996259	**LysoPC (16:0)**	2
	523.7955@6.9962544		
	522.2939@6.9969144		
	522.7951@6.996832		
	762.478@6.997659		
	770.46@6.997441		
	761.976@6.99754		
	769.9581@6.9974766		
	517.3147@6.9961543		
	506.3191@6.9973693		
	495.3327@6.9956923		
	990.6655@6.9950514		
19	527.312@7.05021	Unknown	4
	527.8146@7.050606		
	535.2997@7.0497603		
20	479.3405@7.1102185	**LysoPC(P-16:0)**	2
21	509.3468@7.1235833	**LysoPC(17:0)**	2
22	602.9412@7.1884513	Unknown	4
	597.4188@7.187734		
	610.9343@7.191048		
	611.4359@7.191536		
23	103.0997@7.241595	**LysoPC(18:0)**	2
	523.364@7.2414613		
	534.8485@7.241308		
	534.3476@7.2410583		
	542.3386@7.2418456		
	542.8411@7.2418675		

	550.3265@7.2414513<br>550.8254@7.2414603		
24	545.3467@7.2500143	Unknown	4
25	452.8025@7.3067145	Unknown	4
26	639.4673@7.4324927<br>638.9663@7.4319897	Unknown	4
27	428.3615@7.4910417	Unknown	4
28	284.1159@7.9399796<br>314.1271@7.94182<br>580.232@7.9397755<br>612.2575@7.940997<br>644.2827@7.9412355	**Bilirubin**	1
