# Supplementary material for: Plasma metabolites associated with colorectal cancer: A discovery‐replication strategy
Source: Int J Cancer. 2019 Feb 14;145(5):1221–31. doi: 10.1002/ijc.32146 (PMC6614008; doi:10.1002/ijc.32146)

Supplementary Figure S1. Workflow and study design. (A) The study population consisted of 268 colorectal cancer patients and 353 controls (individuals not diagnosed with any colorectal malignancy), including an independent discovery (ColoCare Study: n=180 patients/n=153 controls) and replication set (CORSA Study: n=88 patients/n=200 controls). (B) Study workflow including feature finding and data alignment (raw data processing), data processing, data analysis (discovery-replication setting) and identification of replicated features.

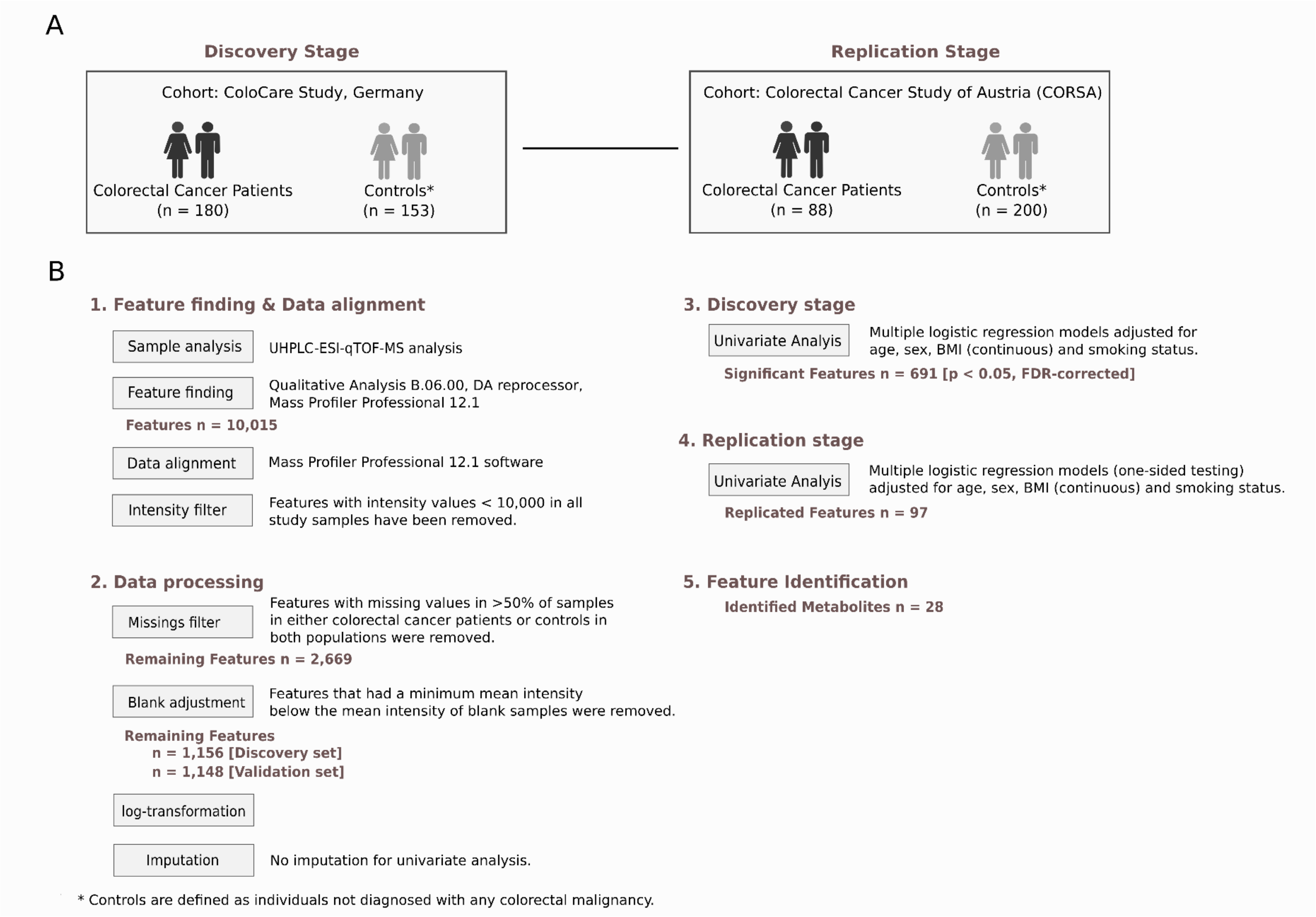

Supplement: Supplementary file 4 — Supplementary Table S3 Spearman correlation coefficients of the 28 metabolites in the discovery and replication stage [file IJC-145-1221-s004.pdf]
