# Supplementary material for: Plasma metabolites associated with colorectal cancer: A discovery‐replication strategy
Source: Int J Cancer. 2019 Feb 14;145(5):1221–31. doi: 10.1002/ijc.32146 (PMC6614008; doi:10.1002/ijc.32146)
Supplement: Supplementary file 5 — Appendix S1: Supporting Information. [file IJC-145-1221-s005.docx]

**Sample preparation and analysis by UHPLC-QTOF-MS**

Samples were randomized across cohort-specific batches and prepared by mixing 20 μL of plasma with 200 μL of acetonitrile. After centrifugation (500 g, 4°C, 10 min), the supernatants were vacuum filtered into 1 ml polypropylene 96-well plates that were sealed and kept at 4°C in the liquid chromatography-mass spectrometry autosampler until analysis (Captiva ND 0.2 μm filter and collection plates, Agilent Technologies, Santa Clara, CA; EPS well plate seals, BioChromato, San Diego, CA). Quality control samples were prepared from a pool of study samples. One blank sample per well plate was prepared along the EDTA plasma samples in an identical manner, only leaving the plasma out of the process.

The auto sampler tray was kept at 4°C and 2 µL of the sample solution was injected on an ACQUITY UHPLC HSS T3 column (2.1 × 100 mm, 1.8 μm; Waters, Milford, MA). Column temperature was maintained at 45°C with a mobile phase flow rate of 0.4 ml/min, consisting of ultrapure water and LC-MS grade methanol, both containing 0.05% (v/v) of formic acid. The gradient profile was as follows: 0–6 min: from 5% to 100% methanol, 6–10.5 min: 100% methanol, 10.5–13 min: 5% methanol.

The mass spectrometer was operated in positive ion mode using the following conditions: drying gas (nitrogen) temperature 175°C and flow 12 L/min, sheath gas temperature 350°C and flow 11 L/min, nebulizer pressure 45 psi, capillary voltage 3500 V, nozzle voltage 300 V, and fragmentor voltage 175 V. Data acquisition was performed using 2 GHz extended dynamic range mode across a mass range of 50–1000. Acquisition rate was 1.67 Hz in centroid mode. Continuous mass axis calibration was performed with two reference ions (*m/z* 121.050873 and *m/z* 922.009798). Data was acquired using MassHunter Acquisition B.05.01 (Agilent Technologies). The analytical run was initiated with priming injections of a quality control sample to achieve stable instrument response, followed by a blank and the study samples.

The processing of acquired metabolomics data was performed using Qualitative Analysis B.06.00, DA reprocessor, and Mass Profiler Professional 12.1 software (Agilent Technologies). Recursive feature finding was employed to find compounds as singly charged ions [M+H]^+^. The initial processing of the data was performed using Qualitative Analysis with the molecular feature extraction algorithm for small molecules. Thresholds for mass and chromatographic peak heights were 1500 and 10000 counts, respectively. Peak spacing tolerance for isotope peaks was 0.0025 *m/z* plus 7 ppm, with the isotope model set to common organic molecules, and with single-ion features also allowed.

**Pre-processing of metabolomics data**

Features found in at least 2% of all samples were combined into a target list, using 0.08 min retention time and 15 ppm + 2 mDa mass windows for alignment. The recursive feature extraction was performed using a find by formula algorithm with match tolerances for the mass and retention time at ±10 ppm and ±0.04 min. Ions were limited to [M+H]^+^.

Alignment was performed with Mass Profiler Professional 12.1 by combining the four compound exchange (.cef) files obtained from the pre-processing of each dataset, using mass and retention time windows of ±15 ppm and ±0.07 min. Successful alignment was facilitated by the high inter and intra-batch reproducibility of retention times, which was assessed by monitoring 13 preselected model compounds in quality control samples, for which retention time variability was less than 0.025 min across all the batches. Features with intensity values below 10,000 in all samples were removed from the analysis.

**Metabolite Identification**

Metabolite identification for UHPLC-QTOF-MS was performed to aim for level 1 identification according to The metabolomics standard initiative.[[47](#_ENREF_47)] Features were clustered by retention time and intensity correlation to help in finding related ions (**Supplementary Table S1**), and m/z values were searched against the human metabolite database (HMDB 4.0) [[48](#_ENREF_48)] using ions [M+H]^+^, [M+Na]^+^, [M-H2O+H]^+^, and [M+2H]^+^, with 7 ppm molecular weight tolerance. Quality of the chromatographic peaks and spectra were inspected, and the plausibility of database candidates was assessed based on retention time, isotope pattern, adduct formation, and neutral losses. Identification was confirmed by analysing pure chemical standards and representative study samples, and by comparing the retention times and MS/MS spectra obtained. When standards were not available, the MS/MS spectra were compared against those in mzCloud [[49](#_ENREF_49)] or Metlin.[[50](#_ENREF_50)] The level of identification was determined as proposed by Sumner *et al.*.[[51](#_ENREF_51)] Details of identification are provided in **Supplementary Table S1**.
