# Supplementary material for: Plasma metabolites associated with colorectal cancer: A discovery‐replication strategy
Source: Int J Cancer. 2019 Feb 14;145(5):1221–31. doi: 10.1002/ijc.32146 (PMC6614008; doi:10.1002/ijc.32146)
Supplement: Supplementary file 3 — Supplementary Table S2 Metabolite identifications of the 97 replicated features [file IJC-145-1221-s003.pdf]

Supplementary Table S3. Spearman correlation coefficients of the 28 metabolites in the discovery and replication stage

DISCOVERY STAGE

	190.0061@	247.0286@	Valine	Leucine	445.8809@	1-methylni	LysoPE(22:	LysoPE(20:	428.3615@	279.1473@	171.0292@	610.9343@	LysoPC(P-1	LyosPC(16:	LysoPC (16	LysoPC(18:	545.3467@	LysoPC(15:	LysoPC(17:	LysoPC(16:	535.2997@	157.1107@	181.1113@	Taurine	Hypoxanth	Bilirubin	452.8025@	638.9663@
190.0061@0.83498704	1	0.307239	0.184357	0.021584	0.2111	0.012261	-0.01502	-0.0641	-0.03051	-0.0877	0.023732	0.164764	0.126671	0.187158	0.157254	0.09128	-0.09022	0.125495	0.12231	0.130248	0.266656	-0.08583	0.148121	-0.1075	-0.26158	-0.0861	-0.18298	-0.16812
247.0286@3.3104632	0.307239	1	0.5564	0.657926	0.060435	0.151317	-0.10516	0.020636	-0.05294	-0.11663	-0.18254	0.203378	0.184575	0.322421	0.308281	0.212535	0.104184	0.228364	0.205328	0.214962	0.311102	0.023298	0.270725	-0.12782	-0.07108	0.299421	0.091799	0.124381
Valine	0.184357	0.5564	1	0.727478	0.259674	0.184437	-0.05271	-0.0137	0.101936	-0.04903	-0.17847	0.218635	0.161193	0.274216	0.268085	0.226298	0.09746	0.155045	0.152446	0.115707	0.258841	-0.00739	0.132801	0.046268	0.112848	0.177797	0.030883	0.017799
Leucine	0.021584	0.657926	0.727478	1	0.270223	0.18409	-0.03564	0.036877	0.007352	-0.07674	-0.20384	0.126002	0.123715	0.280262	0.245622	0.185352	0.204674	0.181499	0.184949	0.112863	0.112162	0.068146	0.139446	0.131575	0.123646	0.371179	0.199648	0.194979
445.8809@0.56495804	0.2111	0.060435	0.259674	0.270223	1	0.229472	0.097458	0.051285	0.024388	-0.08807	-0.03902	0.143593	0.101186	0.058384	0.070544	0.123708	0.163651	0.090003	0.125614	-0.01391	0.052827	0.069226	0.101536	0.049205	-0.17202	0.239351	0.024185	0.033749
1-methylnicotinamide	0.012261	0.151317	0.184437	0.18409	0.229472	1	-0.11146	-0.11125	-0.06346	-0.08779	-0.14316	-0.05258	-0.08935	-0.10005	-0.08454	-0.10152	-0.05708	-0.13366	-0.16585	-0.0293	-0.07235	0.069965	0.117933	0.110192	-0.06798	0.271928	-0.04489	-0.09413
LysoPE(22:6)	-0.01502	-0.10516	-0.05271	-0.03564	0.097458	-0.11146	1	0.53244	0.117366	0.214843	0.223783	0.007817	0.070593	0.086117	0.17008	0.03888	0.135484	-0.01287	0.028477	0.082539	0.027853	0.028572	-0.17074	0.089238	0.248498	-0.04764	-0.06378	0.013008
LysoPE(20:4)	-0.0641	0.020636	-0.0137	0.036877	0.051285	-0.11125	0.53244	1	0.131608	0.041725	0.013878	0.120571	0.157746	0.169363	0.245843	0.163896	0.234412	-0.03572	0.031117	0.255599	0.13991	-0.01725	0.007825	-0.00122	0.158486	0.035616	0.003664	0.149618
428.3615@7.4910417	-0.03051	-0.05294	0.101936	0.007352	0.024388	-0.06346	0.117366	0.131608	1	0.147146	0.122395	-0.00224	0.01395	-0.01651	-0.03172	0.038362	-0.0103	-0.09537	-0.09199	-0.08458	-0.06858	0.059946	-0.08255	-0.05776	0.155711	0.044445	-0.11567	-0.06537
279.1473@2.7668757	-0.0877	-0.11663	-0.04903	-0.07674	-0.08807	-0.08779	0.214843	0.041725	0.147146	1	0.582921	-0.10984	-0.02265	-0.00039	-0.0206	-0.08638	-0.0591	-0.0353	-0.05074	-0.11518	-0.07143	0.08209	-0.0907	0.107143	0.183698	-0.04596	-0.09052	-0.11346
171.0292@2.8578448	0.023732	-0.18254	-0.17847	-0.20384	-0.03902	-0.14316	0.223783	0.013878	0.122395	0.582921	1	-0.10448	-0.0703	-0.04501	-0.06846	-0.13198	-0.1163	-0.02884	-0.07279	-0.09557	-0.08279	-0.01984	-0.1698	0.046681	0.091314	-0.14421	-0.13256	-0.13462
610.9343@7.191048	0.164764	0.203378	0.218635	0.126002	0.143593	-0.05258	0.007817	0.120571	-0.00224	-0.10984	-0.10448	1	0.601606	0.683061	0.662562	0.75096	0.613421	0.473609	0.596438	0.343357	0.571647	0.14545	0.257969	0.002933	0.027332	0.106424	0.405184	0.558157
LysoPC(P-16:0)	0.126671	0.184575	0.161193	0.123715	0.101186	-0.08935	0.070593	0.157746	0.01395	-0.02265	-0.0703	0.601606	1	0.702842	0.771343	0.791325	0.67989	0.564317	0.70241	0.440059	0.627419	0.164544	0.218373	0.04902	0.084196	0.213877	0.333732	0.514644
LyosPC(16:0) isomer	0.187158	0.322421	0.274216	0.280262	0.058384	-0.10005	0.086117	0.169363	-0.01651	-0.00039	-0.04501	0.683061	0.702842	1	0.898525	0.826298	0.714291	0.630625	0.678858	0.628665	0.63264	0.103921	0.270814	0.108025	0.125145	0.172985	0.433044	0.603384
LysoPC (16:0)	0.157254	0.308281	0.268085	0.245622	0.070544	-0.08454	0.17008	0.245843	-0.03172	-0.0206	-0.06846	0.662562	0.771343	0.898525	1	0.84376	0.707388	0.649762	0.723717	0.652944	0.74899	0.145455	0.251238	0.071781	0.198857	0.100395	0.302648	0.511255
LysoPC(18:0)	0.09128	0.212535	0.226298	0.185352	0.123708	-0.10152	0.03888	0.163896	0.038362	-0.08638	-0.13198	0.75096	0.791325	0.826298	0.84376	1	0.894095	0.614678	0.789655	0.478466	0.646896	0.168454	0.29277	0.069956	0.080905	0.154313	0.492279	0.675754
545.3467@7.2500143	-0.09022	0.104184	0.09746	0.204674	0.163651	-0.05708	0.135484	0.234412	-0.0103	-0.0591	-0.1163	0.613421	0.67989	0.714291	0.707388	0.894095	1	0.512999	0.689429	0.408185	0.381982	0.190277	0.208734	0.14482	0.103196	0.270264	0.673426	0.814912
LysoPC(15:0)	0.125495	0.228364	0.155045	0.181499	0.090003	-0.13366	-0.01287	-0.03572	-0.09537	-0.0353	-0.02884	0.473609	0.564317	0.630625	0.649762	0.614678	0.512999	1	0.894002	0.555858	0.505071	0.254828	0.277441	0.011246	0.034187	0.155225	0.276086	0.443424
LysoPC(17:0)	0.12231	0.205328	0.152446	0.184949	0.125614	-0.16585	0.028477	0.031117	-0.09199	-0.05074	-0.07279	0.596438	0.70241	0.678858	0.723717	0.789655	0.689429	0.894002	1	0.473992	0.574892	0.300331	0.259835	0.064186	0.019211	0.188251	0.372399	0.558171
LysoPC(16:1)	0.130248	0.214962	0.115707	0.112863	-0.01391	-0.0293	0.082539	0.255599	-0.08458	-0.11518	-0.09557	0.343357	0.440059	0.628665	0.652944	0.478466	0.408185	0.555858	0.473992	1	0.542199	0.135552	0.274487	0.01517	0.103995	0.058069	0.192563	0.339011
535.2997@7.0497603	0.266656	0.311102	0.258841	0.112162	0.052827	-0.07235	0.027853	0.13991	-0.06858	-0.07143	-0.08279	0.571647	0.627419	0.63264	0.74899	0.646896	0.381982	0.505071	0.574892	0.542199	1	0.100505	0.333007	0.03949	0.134185	-0.0473	-0.04415	0.188989
157.1107@0.8299292	-0.08583	0.023298	-0.00739	0.068146	0.069226	0.069965	0.028572	-0.01725	0.059946	0.08209	-0.01984	0.14545	0.164544	0.103921	0.145455	0.168454	0.190277	0.254828	0.300331	0.135552	0.100505	1	0.217802	0.200437	0.108964	0.186066	0.08713	0.172358
181.1113@5.475981	0.148121	0.270725	0.132801	0.139446	0.101536	0.117933	-0.17074	0.007825	-0.08255	-0.0907	-0.1698	0.257969	0.218373	0.270814	0.251238	0.29277	0.208734	0.277441	0.259835	0.274487	0.333007	0.217802	1	-0.0026	-0.14378	0.166339	0.128734	0.214641
Taurine	-0.1075	-0.12782	0.046268	0.131575	0.049205	0.110192	0.089238	-0.00122	-0.05776	0.107143	0.046681	0.002933	0.04902	0.108025	0.071781	0.069956	0.14482	0.011246	0.064186	0.01517	0.03949	0.200437	-0.0026	1	0.397214	0.058939	0.032283	0.070657
Hypoxanthine	-0.26158	-0.07108	0.112848	0.123646	-0.17202	-0.06798	0.248498	0.158486	0.155711	0.183698	0.091314	0.027332	0.084196	0.125145	0.198857	0.080905	0.103196	0.034187	0.019211	0.103995	0.134185	0.108964	-0.14378	0.397214	1	-0.02204	-0.03791	0.0226
Bilirubin	-0.0861	0.299421	0.177797	0.371179	0.239351	0.271928	-0.04764	0.035616	0.044445	-0.04596	-0.14421	0.106424	0.213877	0.172985	0.100395	0.154313	0.270264	0.155225	0.188251	0.058069	-0.0473	0.186066	0.166339	0.058939	-0.02204	1	0.33797	0.353975
452.8025@7.3067145	-0.18298	0.091799	0.030883	0.199648	0.024185	-0.04489	-0.06378	0.003664	-0.11567	-0.09052	-0.13256	0.405184	0.333732	0.433044	0.302648	0.492279	0.673426	0.276086	0.372399	0.192563	-0.04415	0.08713	0.128734	0.032283	-0.03791	0.33797	1	0.900212
638.9663@7.4319897	-0.16812	0.124381	0.017799	0.194979	0.033749	-0.09413	0.013008	0.149618	-0.06537	-0.11346	-0.13462	0.558157	0.514644	0.603384	0.511255	0.675754	0.814912	0.443424	0.558171	0.339011	0.188989	0.172358	0.214641	0.070657	0.0226	0.353975	0.900212	1

REPLICATION STAGE

	190.0061@	247.0286@	Valine	Leucine	445.8809@	1-methylni	LysoPE(22:	LysoPE(20:	428.3615@	279.1473@	171.0292@	610.9343@	LysoPC(P-1	LyosPC(16:	LysoPC (16	LysoPC(18:	545.3467@	LysoPC(15:	LysoPC(17:	LysoPC(16:	535.2997@	157.1107@	181.1113@	Taurine	Hypoxanth	Bilirubin	452.8025@	638.9663@
190.0061@0.83498704	1	0.098092	0.050408	-0.05996	0.214693	-0.03574	0.015995	-0.0423	-0.18424	-0.19443	-0.0495	0.087555	0.090965	0.216003	0.228239	0.136396	0.076624	0.167161	0.187657	0.147578	0.211003	0.000402	-0.04519	-0.23862	0.021645	-0.07924	-0.00072	0.035465
247.0286@3.3104632	0.098092	1	0.562386	0.69084	0.269853	0.232648	0.070452	0.040585	-0.13626	-0.24215	-0.2278	0.291258	0.229998	0.236591	0.353303	0.246579	0.268534	0.15984	0.179658	0.118467	0.344104	0.04858	0.196766	-0.08487	0.025706	0.322099	0.157402	0.187907
Valine	0.050408	0.562386	1	0.778976	0.448278	0.160666	0.106694	0.121058	-0.01784	-0.05198	-0.11374	0.430366	0.3318	0.325541	0.427587	0.402458	0.334745	0.305176	0.332131	0.238678	0.379633	0.147297	0.14925	0.000309	0.142923	0.307357	0.295797	0.318261
Leucine	-0.05996	0.69084	0.778976	1	0.458815	0.152487	0.084994	0.15949	-0.02693	-0.04242	-0.05202	0.386957	0.302073	0.342675	0.399919	0.346342	0.344186	0.236165	0.275146	0.149149	0.359693	0.2306	0.178337	-0.00694	0.112996	0.361534	0.287261	0.313168
445.8809@0.56495804	0.214693	0.269853	0.448278	0.458815	1	0.106396	0.273271	0.360181	-0.09279	0.102055	-0.00426	0.535866	0.492465	0.464892	0.528584	0.519238	0.377007	0.402969	0.462247	0.366709	0.520854	0.199349	0.117597	-0.01818	0.078708	0.196931	0.277506	0.358986
1-methylnicotinamide	-0.03574	0.232648	0.160666	0.152487	0.106396	1	-0.02375	-0.12612	-0.07421	-0.23252	-0.24355	0.115883	0.102313	0.062859	0.086974	0.094608	0.163304	0.158049	0.126818	0.055515	0.107648	0.071573	0.100589	-0.08292	-0.1062	0.189099	0.082723	0.087685
LysoPE(22:6)	0.015995	0.070452	0.106694	0.084994	0.273271	-0.02375	1	0.57896	0.065462	0.158938	0.081825	0.228998	0.208682	0.223966	0.276479	0.198347	0.244995	0.277001	0.292663	0.321575	0.31037	0.148818	-0.07711	0.181138	0.128063	0.06131	0.120158	0.12414
LysoPE(20:4)	-0.0423	0.040585	0.121058	0.15949	0.360181	-0.12612	0.57896	1	-0.03146	0.223221	0.126535	0.376276	0.345138	0.338243	0.372116	0.355654	0.330749	0.217289	0.285102	0.403647	0.471515	0.253951	0.105151	0.099366	0.155272	0.124421	0.192546	0.26231
428.3615@7.4910417	-0.18424	-0.13626	-0.01784	-0.02693	-0.09279	-0.07421	0.065462	-0.03146	1	0.28436	0.283427	-0.14665	-0.21207	-0.33056	-0.24303	-0.16637	-0.14974	-0.15977	-0.18957	-0.21154	-0.1746	-0.01854	-0.18842	0.235351	0.115148	-0.2099	-0.13946	-0.17231
279.1473@2.7668757	-0.19443	-0.24215	-0.05198	-0.04242	0.102055	-0.23252	0.158938	0.223221	0.28436	1	0.582613	-0.03386	-0.02241	-0.02652	-0.08384	-0.03032	-0.19428	-0.0234	-0.0196	-0.06742	-0.04877	0.051139	-0.05709	0.273121	-0.09739	-0.25065	-0.06567	-0.09403
171.0292@2.8578448	-0.0495	-0.2278	-0.11374	-0.05202	-0.00426	-0.24355	0.081825	0.126535	0.283427	0.582613	1	-0.17733	-0.14457	-0.09169	-0.17557	-0.1942	-0.26338	-0.19252	-0.2027	-0.17522	-0.14128	-0.05768	-0.06954	0.228019	0.051512	-0.25497	-0.19715	-0.26467
610.9343@7.191048	0.087555	0.291258	0.430366	0.386957	0.535866	0.115883	0.228998	0.376276	-0.14665	-0.03386	-0.17733	1	0.79097	0.822322	0.850139	0.910618	0.650393	0.580008	0.686368	0.635636	0.765996	0.208072	0.202525	-0.09798	0.141586	0.329591	0.779627	0.802294
LysoPC(P-16:0)	0.090965	0.229998	0.3318	0.302073	0.492465	0.102313	0.208682	0.345138	-0.21207	-0.02241	-0.14457	0.79097	1	0.795247	0.853234	0.857328	0.600841	0.60809	0.737659	0.588121	0.7404	0.155421	0.161733	-0.1084	0.064489	0.363564	0.623114	0.709913
LyosPC(16:0) isomer	0.216003	0.236591	0.325541	0.342675	0.464892	0.062859	0.223966	0.338243	-0.33056	-0.02652	-0.09169	0.822322	0.795247	1	0.927154	0.849922	0.618201	0.640248	0.723491	0.657876	0.772822	0.140504	0.146314	-0.15822	0.077805	0.349271	0.711257	0.750365
LysoPC (16:0)	0.228239	0.353303	0.427587	0.399919	0.528584	0.086974	0.276479	0.372116	-0.24303	-0.08384	-0.17557	0.850139	0.853234	0.927154	1	0.911218	0.647976	0.668122	0.754354	0.706918	0.827605	0.148316	0.192895	-0.15279	0.119668	0.317	0.673356	0.734782
LysoPC(18:0)	0.136396	0.246579	0.402458	0.346342	0.519238	0.094608	0.198347	0.355654	-0.16637	-0.03032	-0.1942	0.910618	0.857328	0.849922	0.911218	1	0.684379	0.619442	0.745083	0.653337	0.774612	0.166097	0.170876	-0.11572	0.113943	0.278606	0.718339	0.809889
545.3467@7.2500143	0.076624	0.268534	0.334745	0.344186	0.377007	0.163304	0.244995	0.330749	-0.14974	-0.19428	-0.26338	0.650393	0.600841	0.618201	0.647976	0.684379	1	0.420987	0.531788	0.409801	0.51872	0.176938	0.071136	-0.0732	0.177329	0.31378	0.605877	0.650404
LysoPC(15:0)	0.167161	0.15984	0.305176	0.236165	0.402969	0.158049	0.277001	0.217289	-0.15977	-0.0234	-0.19252	0.580008	0.60809	0.640248	0.668122	0.619442	0.420987	1	0.912966	0.550768	0.578659	0.164333	0.120746	-0.11009	0.038771	0.269	0.416095	0.513509
LysoPC(17:0)	0.187657	0.179658	0.332131	0.275146	0.462247	0.126818	0.292663	0.285102	-0.18957	-0.0196	-0.2027	0.686368	0.737659	0.723491	0.754354	0.745083	0.531788	0.912966	1	0.53147	0.669936	0.20689	0.162133	-0.15119	0.001771	0.276424	0.502002	0.608097
LysoPC(16:1)	0.147578	0.118467	0.238678	0.149149	0.366709	0.055515	0.321575	0.403647	-0.21154	-0.06742	-0.17522	0.635636	0.588121	0.657876	0.706918	0.653337	0.409801	0.550768	0.53147	1	0.649937	0.098591	0.038844	-0.09766	0.159323	0.267793	0.496519	0.559359
535.2997@7.0497603	0.211003	0.344104	0.379633	0.359693	0.520854	0.107648	0.31037	0.471515	-0.1746	-0.04877	-0.14128	0.765996	0.7404	0.772822	0.827605	0.774612	0.51872	0.578659	0.669936	0.649937	1	0.162601	0.243283	-0.09633	0.093858	0.298297	0.496632	0.559738
157.1107@0.8299292	0.000402	0.04858	0.147297	0.2306	0.199349	0.071573	0.148818	0.253951	-0.01854	0.051139	-0.05768	0.208072	0.155421	0.140504	0.148316	0.166097	0.176938	0.164333	0.20689	0.098591	0.162601	1	0.073208	0.038715	-0.02584	0.173194	0.122087	0.140547
181.1113@5.475981	-0.04519	0.196766	0.14925	0.178337	0.117597	0.100589	-0.07711	0.105151	-0.18842	-0.05709	-0.06954	0.202525	0.161733	0.146314	0.192895	0.170876	0.071136	0.120746	0.162133	0.038844	0.243283	0.073208	1	0.01773	0.049531	0.108024	0.103582	0.124551
Taurine	-0.23862	-0.08487	0.000309	-0.00694	-0.01818	-0.08292	0.181138	0.099366	0.235351	0.273121	0.228019	-0.09798	-0.1084	-0.15822	-0.15279	-0.11572	-0.0732	-0.11009	-0.15119	-0.09766	-0.09633	0.038715	0.01773	1	0.206528	-0.13509	-0.12887	-0.15488
Hypoxanthine	0.021645	0.025706	0.142923	0.112996	0.078708	-0.1062	0.128063	0.155272	0.115148	-0.09739	0.051512	0.141586	0.064489	0.077805	0.119668	0.113943	0.177329	0.038771	0.001771	0.159323	0.093858	-0.02584	0.049531	0.206528	1	0.074789	0.151032	0.086294
Bilirubin	-0.07924	0.322099	0.307357	0.361534	0.196931	0.189099	0.06131	0.124421	-0.2099	-0.25065	-0.25497	0.329591	0.363564	0.349271	0.317	0.278606	0.31378	0.269	0.276424	0.267793	0.298297	0.173194	0.108024	-0.13509	0.074789	1	0.388136	0.397222
452.8025@7.3067145	-0.00072	0.157402	0.295797	0.287261	0.277506	0.082723	0.120158	0.192546	-0.13946	-0.06567	-0.19715	0.779627	0.623114	0.711257	0.673356	0.718339	0.605877	0.416095	0.502002	0.496519	0.496632	0.122087	0.103582	-0.12887	0.151032	0.388136	1	0.871556
638.9663@7.4319897	0.035465	0.187907	0.318261	0.313168	0.358986	0.087685	0.12414	0.26231	-0.17231	-0.09403	-0.26467	0.802294	0.709913	0.750365	0.734782	0.809889	0.650404	0.513509	0.608097	0.559359	0.559738	0.140547	0.124551	-0.15488	0.086294	0.397222	0.871556	1
